# Re-analysis of archaeobotanical remains from pre- and early agricultural sites provides no evidence for a narrowing of the wild plant food spectrum during the origins of agriculture in southwest Asia

**DOI:** 10.1007/s00334-018-0702-y

**Published:** 2018-11-17

**Authors:** Michael Wallace, Glynis Jones, Michael Charles, Emily Forster, Eleanor Stillman, Vincent Bonhomme, Alexandra Livarda, Colin P. Osborne, Mark Rees, Georg Frenck, Catherine Preece

**Affiliations:** 1 Department of Archaeology, University of Sheffield, Minalloy House, 10-16 Regent Street, Sheffield, S1 3NJ UK; 2 Department of Archaeology, University of Oxford, 36 Beaumont Street, Oxford, OX1 2PG UK; 3 School of Mathematics and Statistics, University of Sheffield, Hicks Building, Hounsfield Road, Sheffield, S3 7RH UK; 4 Institut des Sciences de l’Evolution-Montpellier (ISEM-UMR 5554), Equipe Dynamique de la Biodiversité, Anthropo-écologie, Université de Montpellier, 34095 Montpellier Cedex 2, France; 5 Department of Classics and Archaeology, University of Nottingham, University Park, Nottingham, NG7 2RD UK; 6 Department of Animal and Plant Sciences, University of Sheffield, Alfred Denny Building, Western Bank, Sheffield, S10 2TN UK; 7 Department of Ecology, University of Innsbruck, Technikerstraße 25, 6020 Innsbruck, Austria; 8 CREAF, Campus de Bellaterra (UAB), Edifici C, 08193 Cerdanyola del Vallès, Spain

**Keywords:** Archaeobotany, Neolithic, Pre-Pottery Neolithic, Wild plant foods, Wild plants, Broad spectrum

## Abstract

Archaeobotanical evidence from southwest Asia is often interpreted as showing that the spectrum of wild plant foods narrowed during the origins of agriculture, but it has long been acknowledged that the recognition of wild plants as foods is problematic. Here, we systematically combine compositional and contextual evidence to recognise the wild plants for which there is strong evidence of their deliberate collection as food at pre-agricultural and early agricultural sites across southwest Asia. Through sample-by-sample analysis of archaeobotanical remains, a robust link is established between the archaeological evidence and its interpretation in terms of food use, which permits a re-evaluation of the evidence for the exploitation of a broad spectrum of wild plant foods at pre-agricultural sites, and the extent to which this changed during the development of early agriculture. Our results show that relatively few of the wild taxa found at pre- and early agricultural sites can be confidently recognised as contributing to the human diet, and we found no evidence for a narrowing of the plant food spectrum during the adoption of agriculture. This has implications for how we understand the processes leading to the domestication of crops, and points towards a mutualistic relationship between people and plants as a driving force during the development of agriculture.

**Electronic supplementary material:**

The online version of this article (10.1007/s00334-018-0702-y) contains supplementary material, which is available to authorized users.

## Introduction

### The collection of wild plant foods

The emergence of agriculture in the Pre-Pottery Neolithic (PPN) of southwest Asia marked a major change in human subsistence strategies, with a shift from communities based on the gathering of wild plants to those reliant primarily on the cultivation of eight founder crops (einkorn, emmer, barley, lentil, pea, chickpea, bitter vetch and flax). It is widely accepted that a broad spectrum of plant foods was exploited by late Palaeolithic hunter-gatherers and that there was a narrowing of this spectrum with the advent of domesticated crops and the emergence of agriculture (e.g. Weiss et al. 2004; Savard et al. 2006; Willcox et al. 2008; Colledge and Conolly 2010).

The rise and fall of a broad spectrum of food resources has been linked to notions of optimal foraging theory whereby, in the face of resource depletion due to population increase and/or environmental change, foragers increasingly exploited lower ranked resources which subsequently declined in importance with the advent of higher ranking resources such as domesticated plants and animals (Flannery 1969; Stiner et al. 2000; Stiner 2001; Stiner and Munro 2002; Winterhalder and Kennett 2006; Gremillion et al. 2014). Following this view, Weiss et al. (2004) have argued that a broad range of small-seeded grasses contributed to the forager diet in the Upper Palaeolithic but that their contribution declined in comparison with that of wild cereals until the PPNA (early PPN), after which their significance was negligible. They see the flourishing of a broad range of plant foods in the Upper Palaeolithic as indicating a temporary switch to low-ranked foods due to pressure on food resources.

There are several reasons, however, why the exploitation of a broad spectrum of foods might persist, including buffering against the risk of food shortages or a reluctance to abandon culturally preferred wild foods. An alternative view is that, rather than reflecting an increased use of lower ranked taxa, the exploitation of a broad spectrum of foods was an opportunistic response to plentiful and diverse resource availability (Smith 2007, 2011a, b), which facilitated more permanent settlement, and included managed or domesticated plants alongside collected wild foods (Zeder 2012, 2015, 2016). Savard et al. (2006) provide some support for this view. Using a different quantification method to that used by Weiss et al. (2004), they suggest that the use of small-seeded grasses did not fall sharply after the Epipalaeolithic period in the eastern Fertile Crescent, but rather that these grasses or other non-grass species continued to form a significant component of the diet throughout the PPNA, suggesting an opportunistic approach to the collection of wild plant foods. Though contradictory, the arguments of both Weiss et al. (2004) and Savard et al. (2006) are based on the assumption that the plant remains present on early sites were primarily collected as food.

However, it has long been acknowledged that the recognition of wild plants as foods is problematic (e.g. Dennell 1976; van Zeist and Bakker-Heeres 1985; Willcox et al. 2008). First, there are other means by which seeds from wild plants may have inadvertently arrived on archaeological sites, including their unintentional collection together with wild food plants (e.g. Savard et al. 2003, p 102) or with woody material (Arranz-Otaegui et al. 2018), their arrival on site as weeds of crops (e.g. Hillman 1984, 2000), or their inclusion in animal dung (e.g. Miller and Smart 1984; Miller 1984, 1996; Spengler 2018). Secondly, even when it can be established that a plant was deliberately brought onto site, there are numerous reasons other than its use as food why it may have been collected, e.g. as building material, bedding or fuel. The aims of this paper are therefore (1) to distinguish the suite of wild plants for which there is strong evidence that they were (a) deliberately collected and (b) were intended to be used as food by pre-agricultural or early agricultural communities, and (2) to re-evaluate the evidence for the exploitation of a broad spectrum of wild plant foods, and the extent to which this changed during the development of early agriculture.

### Contextual and compositional evidence

Two criteria have been advocated by archaeobotanists as a means of distinguishing the deliberate collection of wild plants from their unintentional arrival on site: archaeological context, such as in a storage pit or vessel (van Zeist and Bakker-Heeres 1985, p 247) and high concentrations of seeds of the same type (e.g. Willcox et al. 2008, p 317). These criteria have been used in conjunction as in the case of concentrations of seeds in plaster bins at Çatalhöyük (Bogaard et al. 2009; Twiss et al. 2009) and Hacılar (Helbæk 1970), in proximity to food processing areas at Ohalo II (Weiss et al. 2008) and Jerf el Ahmar (Willcox 2002), and beneath a granary at Gilgal (Weiss et al. 2006). Arranz-Otaegui et al. (2016) have recently commented, however, on the overall lack of detailed analyses of sample composition of PPN archaeobotanical assemblages and the poor recording of contextual associations between plant remains and archaeological features. Here we address these issues by considering both the botanical composition of individual samples and, where known, their archaeological context, in order to achieve our first aim of establishing a robust link between the archaeological evidence and its interpretation in terms of deliberate collection and use. Approaches based on the overall frequency/ubiquity or abundance of wild species in a site (or period) assemblage are unable to achieve this, and can merely indicate the availability of wild species. We therefore consider the sample-level approach adopted here as a necessary precursor to our second aim, an investigation of the spectrum of wild plant foods at PPN sites and changes in wild food use through time.

Contexts associated with storage or processing are most likely to indicate the deliberate collection and use of plants. Storage contexts may include ceramic vessels, clay bins, pits and receptacles composed of more perishable materials, such as reed baskets (Hillman et al. 1989; Özdoğan 2009, p 23). Pots and bins are good storage indicators but are rarely found in the PPN, and even these may have received secondary deposits of plant material after they had gone out of use as storage devices. Pits are ambiguous indicators of storage, as they were often used for other purposes such as disposal of refuse. Storage in perishable containers is usually only identifiable from concentrations of plant material (usually in burnt destruction deposits), and such concentrations are particularly indicative of storage if found in association with (e.g. in the same room as) less perishable storage containers, as at Jerf el Ahmar (Willcox 2002) and Çatalhöyük (Bogaard et al. 2009; Twiss et al. 2009). Processing of plants for use may be indicated by association with processing facilities or by proximity to hearths or ovens in which they may have been accidentally charred. For all contexts, however, re-use and the introduction of secondary plant material can only be excluded through analysis of botanical composition.

Two compositional characteristics of an archaeobotanical sample that have been used to indicate storage are density and purity (Dennell 1976; Jones 1991). The greater the density of plant remains [number of plant items − usually diaspores (hereafter referred to as seeds) per unit volume of sediment], the more likely it is that they were deposited as a single entity. A sample of high purity (e.g. dominated by one species) is likely to be the result of a depositional event relating to the dominant species within the sample. The combination of high density and high purity is thus a good indication of storage of the dominant species, and concentrations of many hundreds of seeds of the same species are often considered unambiguous indicators of storage. Even in such extreme cases, samples are unlikely to be absolutely pure due to the presence of contaminants introduced during collection, storage or disturbance, or the storage of several species together. Some plant material originally deposited in storage contexts and so originally dense and pure may have been subsequently disturbed or re-deposited, resulting in decreased density and purity. The recognition of such store-derived samples may thus increase the likelihood of successfully recognising deliberately collected plant taxa, as could the identification of processing for use.

## Methodology

### Archaeobotanical database

**Fig. 1:**
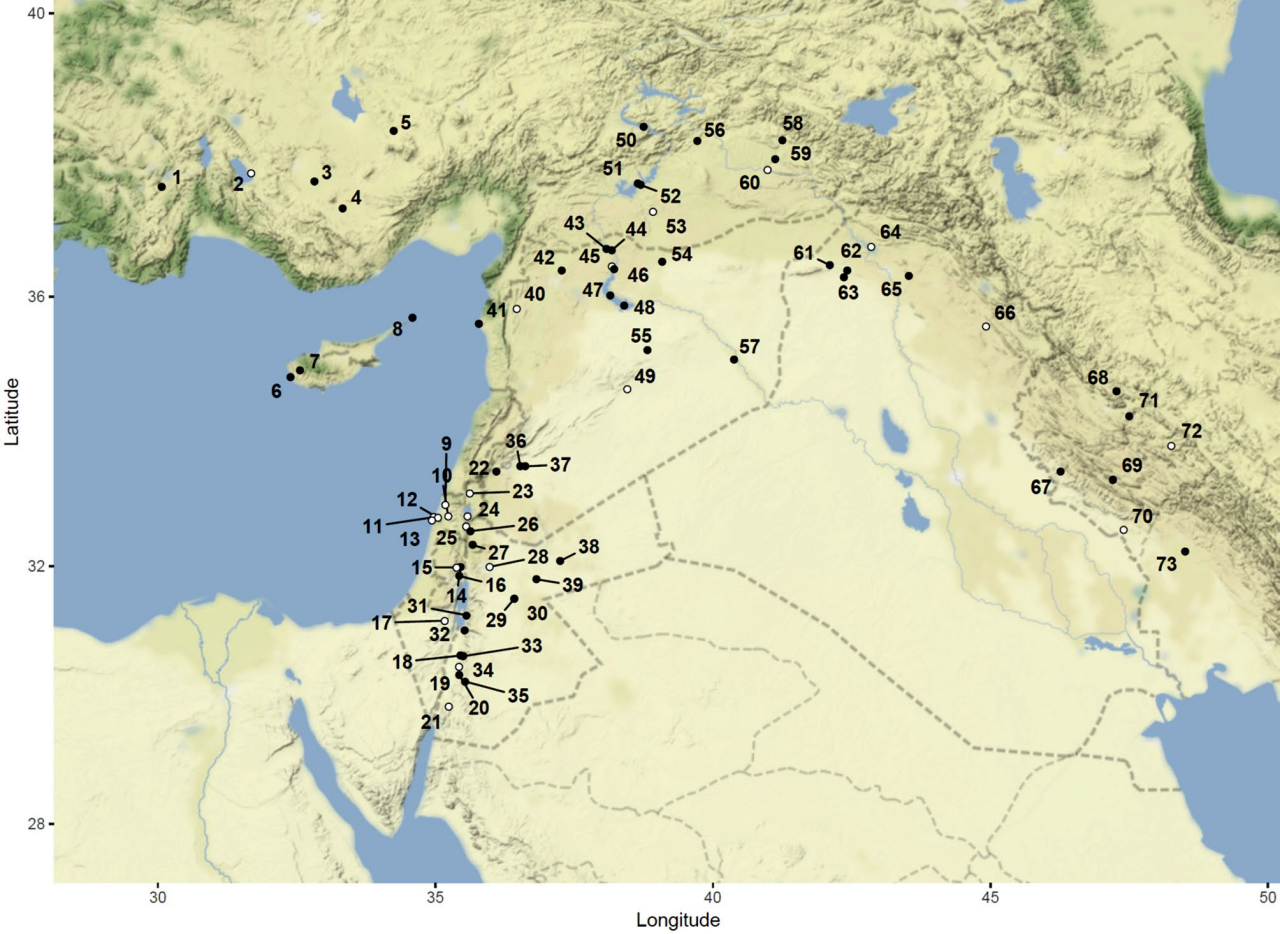
Map of the Pre-Pottery Neolithic sites in southwest Asia with archaeobotanical remains included in the database. Black symbols indicate sites for which sample-level data was available; white symbols indicate sites for which sample-level data was unavailable. Central Anatolian sites—1: Hacılar, 2: Erbaba, 3: Çatalhöyük East, 4: Can Hasan III and 5: Aşikli Höyük. Cypriot sites—6: Mylouthkia, 7: Ais Yiorkis and 8: Kastros. Southern Levantine sites—9: Hayonim Cave, 10: Yiftahel, 11: Nahal Oren, 12: Kebara Cave, 13: Atlit-Yam, 14: Gilgal, 15: Netiv Hagdud, 16: Jericho, 17: Nahal Hemar, 18: Wadi Faynan 16, 19: Shkarat Msaied, 20: Beidha, 21: Ayn Abu Nukhayla, 22: Tell Ramad, 23: Gesher Benot Yaaqov, 24: Ohalo II, 25: Gesher, 26: Wadi al-Hammeh 27, 27: Iraq ed-Dubb, 28: Ain Ghazal, 29: Wadi el-Jilat 13, 30: Wadi el-Jilat 6 & 7, 31: Zahrat adh-Dhra 2, 32: el-Hemmeh, 33: Wadi Fidan A, 34: Wadi Fidan C, 35: Basta I, 36: Tell Ghoraifé, 37: Tell Aswad, 38: Dhuweilla and 39: Azraq 31. Northern Levantine sites—40: Tell Ain el-Kerkh, 41: Tell Ras Shamra, 42: Tell Qaramel, 43: Tell Abr, 44: Dj’ade, 45: Halula, 46: Jerf el Ahmar, 47: Mureybet, 48: Abu Hureyra, 49: Douara Cave, 50: Cafer Höyük, 51: Gritille, 52: Nevali Çori, 53: Göbekli Tepe, 54: Tell Sabi Abyad II, 55: El Kowm I & II, 56: Çayönü and 57: Tell Bouqras. Sites of the eastern Fertile Crescent—58: Hallan Çemi, 59: Demirkoy, 60: Kortik Tepe, 61: Tell Maghzaliyeh, 62: Qermez Dere, 63: Yarym Tepe, 64: Nemrik 9, 65: Mlefaat, 66: Jarmo, 67: Chogha Golan, 68: Sheikh-e Abad, 69: Chia Sabz, 70: Tepe Ali Kosh, 71: Ganj Dareh Tepe, 72: Tepe Abdul Hosein and 73: Chogha Bonut

We systematically reviewed the published and, where possible, unpublished archaeobotanical data for all PPN and earlier sites (primarily 12000–5000 cal bc) in southwest Asia (central Anatolia, Cyprus, the southern and northern Levant, and the eastern Fertile Crescent) at which plant remains have been found (75 sites, Fig. 1). Archaeobotanical records of charred plant remains were entered into a database as individual samples in all cases for which sample-level data were available (3,162 samples from 52 sites; see ESM Table A which also lists the sources of data and ESM Text A which provides details of an online version of the database). This provides the finest resolution possible for published records and site archives, and therefore the closest approximation of individual “behavioural episodes” (Jones 1991).

To prepare the database for analysis, it was necessary to standardise both the plant nomenclature and the method of quantification. Taxonomic synonyms were therefore combined to give aggregated counts under a single taxonomic name. For crops, the nomenclature proposed by Miller (1992) was adopted. This standardisation reduced the original 844 taxonomic entries to 698 unique taxa. The most common method of quantification used in the source literature was a count of the number of plant items. Where other quantification methods were used, such as weight, scales of abundance or presence, values were converted to counts as far as possible. Some authors provide conversion factors for converting weight or abundance scales into counts, but in the case of simple presence a count of one was attributed to each reported taxon. Samples for which the data were originally recorded as counts, or where data could be reliably converted to counts, constitute 92% of the total samples.

### Contextual evidence

The quality of contextual information in published reports is highly variable. Detailed contextual descriptions for individual samples are rarely provided (but see Miller 2002; Fairbairn 2007), and it is more common to find key words relating to context. Where possible, we assigned samples to one of eight context categories (containers, burnt destruction deposits, pits, internal fire installations, external burnt areas, refuse deposits, internal spaces and external spaces) as in Table 1. Some of these relate to potential storage (e.g. containers) and some to the disposal of refuse, whilst others are more ambiguous (e.g. internal spaces).

**Table 1:** Context categories and the types of contexts included in each category

Context category	Context types
Containers	Clay bins, ceramic vessels
Burnt destruction deposits	Houses, rooms, internal spaces; with evidence of extensive burning
Pits	Both internally and externally located
Internal fire installations	Ovens, hearths, rake out, interior burnt areas
External burnt areas	Fire-spots, burnt/ash deposits, exterior hearths
Refuse deposits	Middens, dumps, trash/rubbish
Internal spaces	Occupation layers/deposits, floors, houses, rooms; not extensively burnt
External spaces	Not further specified

### Compositional analysis

Sample density could not be used routinely in our analysis because the volume of sediment was often not recorded in archaeobotanical reports. As an alternative, therefore, we used absolute counts of plant seeds. While this is problematic, because total counts are partly determined by sample density but also by the size of the sample taken, seed counts are almost always available. In addition, samples with a total count of less than 50 seeds were excluded from our analyses as they were considered too small to be reliable indicators of deliberate collection (cf. Halstead 1994, where the same numerical cut-off was used to indicate the deliberate cultivation of crops).

Sample purity can be quantified using a diversity index, based on the number of species and the evenness in their abundance. For our purposes, the Simpson index D (Simpson 1949) is appropriate because, unlike the well-known Shannon index (Shannon and Weaver 1948), it is primarily influenced by evenness, such that the number of species making up the minority component of the sample does not substantially alter the score (Morris et al. 2014; see also ESM Fig. A for examples based on synthetic data). On a 0 to 1 scale, the Simpson index assigns a high score to samples with low diversity (high purity) and vice versa. It is given by:

$${\text{D}}={\sum {\left( {\frac{{\text{n}}}{{\text{N}}}} \right)} ^2}$$

where, D = Simpson index, n = count for a particular taxon, N = count for all taxa.

In addition, archaeobotanical material is often identifiable only to genus or family. This may result in several species being included under a single taxonomic identification, giving the semblance of greater purity. Conversely, a sample may appear less pure when the same species is identified at different taxonomic levels as, for example, when some seeds are identified to species and others (usually less well preserved) are identified only to genus. The Simpson index should therefore be taken only as an estimate of a sample’s purity.

### Data analysis

For quantified samples with at least 50 seeds (1,381 samples from 49 sites), we examined the relationship between the count of seeds (as a proxy for density) and the Simpson index (as a estimate of purity), first in order to investigate the relationship of compositional data to archaeological context and, secondly, to derive a measure of the likelihood of deliberate collection. For these purposes, the counts were capped at 10,000 to provide a range with fixed start and end points, and then converted to a scale of 0 to 1 to make them comparable with the Simpson index. The count of 10,000 was selected as the upper limit because this encompasses most of the sample sizes in our database (ESM Fig. B). These raw counts were then transformed by taking logs to the base ten, which gives greater weight to differences in sample size at the lower end of the range, and dividing the log_10_ value by 4 (the number of log_10_ steps between 1 and 10,000) to arrive at a scale of 0 to 1. This can be summarised as follows:

$$C=\frac{{{{\log }_{10}}N}}{4}$$

where, C = normalised logarithmic count, N = raw counts.

For each sample, the new normalised logarithmic count (C) was combined with the Simpson index (D), by calculating the Euclidian distance between a sample and the maximum normalised logarithmic score of 1 (i.e. 10,000 or more seeds) and the maximum Simpson index of 1 (i.e. only one taxon in the sample). This provides an indicator of deliberate collection (DC score), normalised to a scale of 0 to 1, as follows:

$${\text{DC=1}} - \frac{{\sqrt {{{(1 - D)}^2}+{{(1 - C)}^2}} }}{{\sqrt 2 }}$$

## Results

### The relationship of botanical composition to archaeological context

**Fig. 2:**
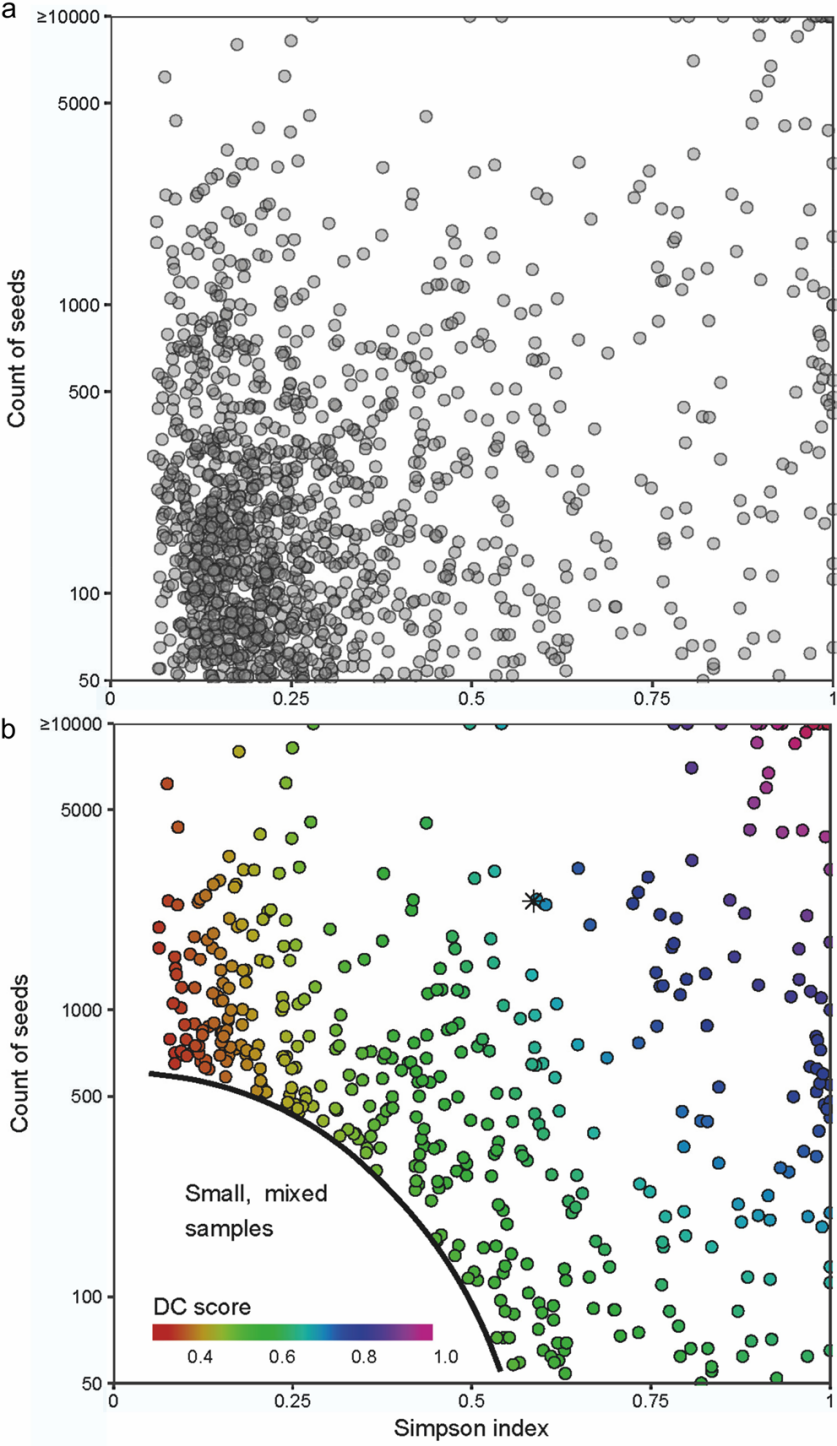
Counts of plant items in archaeobotanical samples plotted on a logarithmic scale against Simpson index. **a** All samples; **b** samples coded by DC score (small and mixed samples—see ESM Equation A—not plotted). The approximate location of the plant remains from square F78c, Floor II, Hut 1 at Ohalo II is plotted as an asterisk in **b**

To investigate the relationship of compositional data to archaeological context, we examined “abundance versus purity” plots of the count of seeds in each sample (plotted on a logarithmic scale) against the Simpson index. Many of the samples are neither rich in plant remains nor compositionally pure, and are therefore concentrated in the lower left part of Fig. 2a. These small mixed samples are least likely to represent deliberate collection and were therefore excluded from further analysis (ESM Equation A, for definition of small and mixed). The resulting dataset (comprising 41 sites and 477 samples) is restricted to samples that are either rich (in the upper part of Fig. 2a, b) or pure (to the right side of Fig. 2a, b) or both (upper right of Fig. 2a, b).

**Fig. 3:**
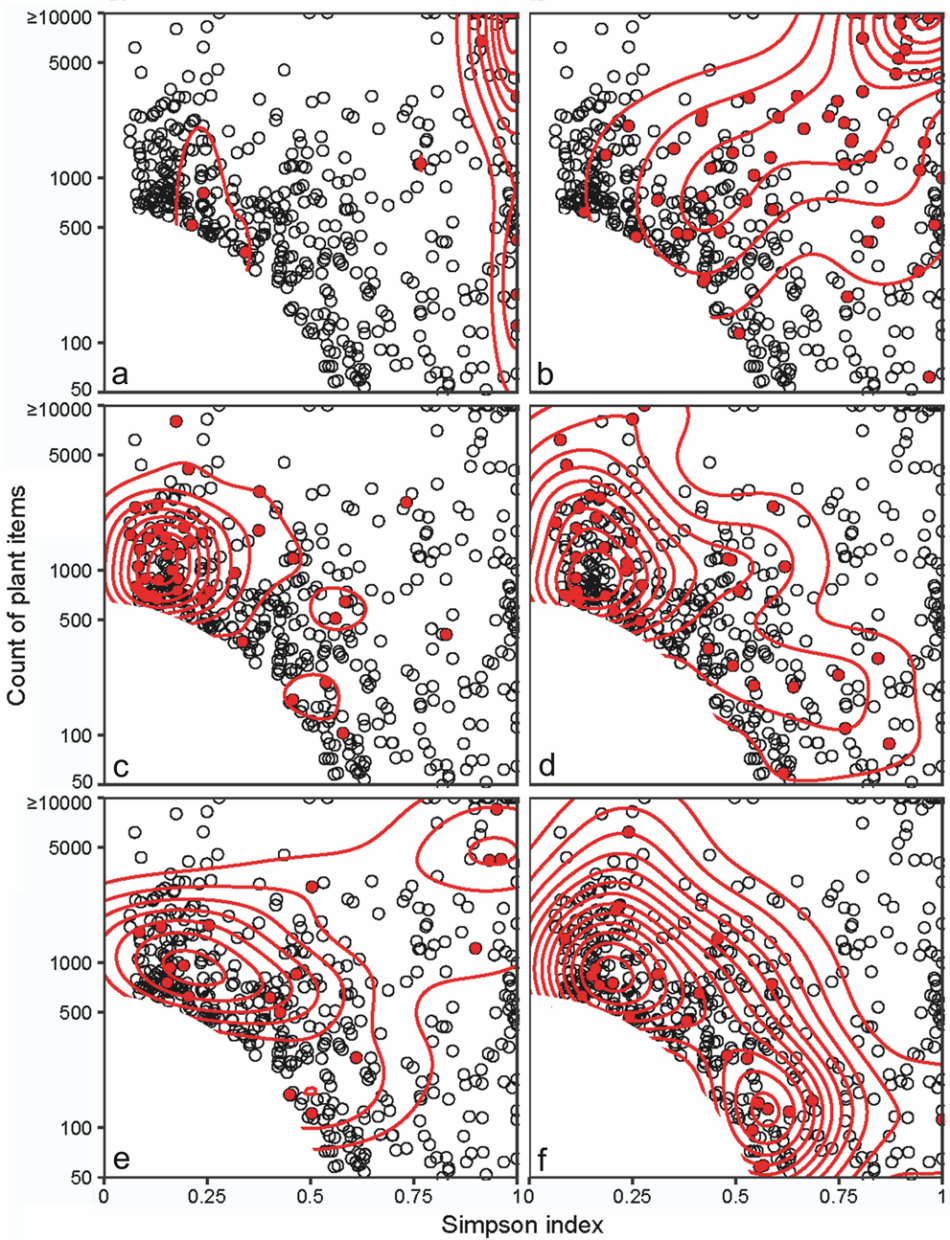
Counts of plant items in archaeobotanical samples plotted on a logarithmic scale against Simpson index (small and mixed samples—see ESM Equation A—not plotted). Red (filled) symbols indicate samples from the following context categories: **a** containers; **b** burnt destruction deposits; **c** refuse deposits; **d** external burnt areas; **e** internal fire installations; **f** pits. Contour lines indicate density estimations for each context category; multiple lines indicate increasing concentrations of samples

To incorporate archaeological context into this compositional plot, samples in the restricted dataset were re-coded according to the eight context categories in Table 1 (see also ESM Table B). It is clear from these plots (Fig. 3 and ESM Fig. C) that some context categories tend to be concentrated in particular areas of the plot. Samples from containers, for example, are primarily concentrated to the right of the plot (Fig. 3a) because of their purity, and especially towards the top right, due their large size. These samples almost certainly represent the remains of stored seed. A group of three samples from containers are more mixed in composition, clustering towards the left of the plot, which may indicate secondary deposition in these containers. Samples from burnt destruction deposits show a similar distribution to those from containers (Fig. 3b), but are rather more dispersed. This is to be expected if they primarily represent seeds stored within a building and charred during its destruction, those samples to the right representing relatively pure concentrations of stored products and those towards the left resulting from mixing either between stored products or with plant material lying on the floor of the building at the time of its destruction.

At the other extreme, samples from refuse deposits tend to be concentrated towards the left of the plot (Fig. 3c), reflecting their generally mixed composition, though some of these samples are very rich in plant remains. This clearly demonstrates that quantity alone is not a sufficient indication of storage. Samples from external burnt areas show a similar distribution to the samples from refuse deposits (Fig. 3d) though some of these are of greater compositional purity, perhaps indicating the processing of plant material involving fire at some of these locations. Internal fire installations are even more widely distributed across the plot (Fig. 3e), with several particularly large pure samples located towards the top right of the plot. All of these are from ovens in burnt destruction levels, so the plant remains probably derive from the final destruction of the building by fire rather than the earlier use of the ovens themselves. Other less specific context categories such as pits (Fig. 3f) and general classifications such as internal or external spaces (ESM Fig. C) also exhibit relatively wide distributions. Based on these results, we adopt a two-stage method for the recognition of wild food plants: first we consider the likelihood that taxa were deliberately collected, and secondly, from amongst the probably collected taxa, we distinguish those likely to have been collected as food from those more likely to have been collected for other purposes. At both stages, the quality of the evidence defines the certainty with which inferences about collection and use can be made and, inevitably, some inferences are more certain than others, as indicated below.

### The recognition of deliberate plant collection

The relationships between sample composition and archaeological context clearly demonstrate that, as expected, likely storage contexts tend to be associated with archaeobotanical samples that are both rich in plant remains and relatively pure in terms of species composition. It is also apparent that abundance of plant remains alone is as likely to be associated with rubbish deposits as with storage contexts. This allows us to assess the likely derivation of archaeobotanical samples using the Deliberate Collection (DC) score (Fig. 2b). The higher the score, the more rich and pure the sample, and the more likely it is to represent storage; the lower the score the more likely it is that the sample represents discarded material. Samples with intermediate DC scores may represent mixed or disturbed stored plant material or the debris of plant processing, though some may also derive from refuse contexts. A concentration of plant remains from the northern part of Hut 1 at Ohalo II, associated with a grinding stone (ESM Table C), has an intermediate DC score (0.57, Fig. 3), which is consistent with its interpretation as the remnants of processing, as supported by starch grain analysis (Piperno et al. 2004; Weiss et al. 2008).

As well as recognising samples that represent deliberate collection, it is also necessary to distinguish which species in these samples were the objects of storage or processing, while not overlooking the possible storage of mixed species or multiple episodes of processing. So, to allow the recognition of up to three deliberately collected species per sample, taxa that comprise at least 30% of a sample indicated as deliberately collected are considered a potential component of the stored or processed product (cf. Halstead 1994, where the same percentage cut-off was used to indicate deliberate cultivation of crop species). Some seeds are identified only to higher taxonomic levels, so all taxa (species, genus or family identifications) that make up ≥ 30% of at least one sample with a relatively high DC score (≥ 0.5), are listed in Table 2 (domesticates and their wild progenitors) or Table 3 (other wild taxa). Whilst the DC scores for each taxon may be based on only one sample (ESM Table D), it nevertheless provides the best single piece of evidence for that taxon’s deliberate collection. Taxa in the upper part of Tables 2 and 3 (from samples with the highest DC scores) are those with the strongest evidence for deliberate collection and storage. Taxa in the lower part of the tables (from samples with progressively lower DC scores) are successively less likely to be from a single stored product, and are more likely to result from processing for use, mixed stored products, or domestic refuse. It should be noted that Tables 2 and 3 do not rank taxa in order of importance, but rather by the quality of the evidence for (and so the likelihood of) their deliberate collection.

**Table 2:** Deliberate collection (DC) scores for domesticated crops and wild progenitors with DC scores ≥ 0.5, grouped by domestication status. ^a^Presumed status only

Taxon	Family	DC
Domesticated crops		
*Triticum monococcum*	Poaceae	1.00
*Lens culinaris*	Fabaceae	0.99
*Hordeum vulgare*	Poaceae	0.98
*Triticum dicoccum*/*monococcum*	Poaceae	0.86
*Vicia ervilia*^a^	Fabaceae	0.81
*Hordeum vulgare* var. *nudum*	Poaceae	0.80
*Hordeum vulgare distichum*	Poaceae	0.75
*Pisum sativum*	Fabaceae	0.74
*Triticum aestivum*/*durum*	Poaceae	0.70
*Hordeum vulgare* var. *nudum*/*spontaneum*	Poaceae	0.67
*Linum usitatissimum*	Linaceae	0.64
*Hordeum vulgare hexastichum*	Poaceae	0.61
*Triticum dicoccum*	Poaceae	0.57
*Triticum spelta*	Poaceae	0.54
Domesticated crops/crop progenitors		
*Lens*^a^	Fabaceae	0.98
*Hordeum vulgare*/*spontaneum*	Poaceae	0.82
*Pisum*/*Vicia*/*Lathyrus*^a^	Fabaceae	0.69
Cereal indeterminate^a^	Poaceae	0.66
*Triticum*^a^	Poaceae	0.57
Crop progenitors		
*Pisum elatius*	Fabaceae	0.91
*Hordeum spontaneum*	Poaceae	0.85
*Triticum boeoticum thaoudar*	Poaceae	0.84
*Triticum*/*Secale*^a^	Poaceae	0.83

**Table 3:**
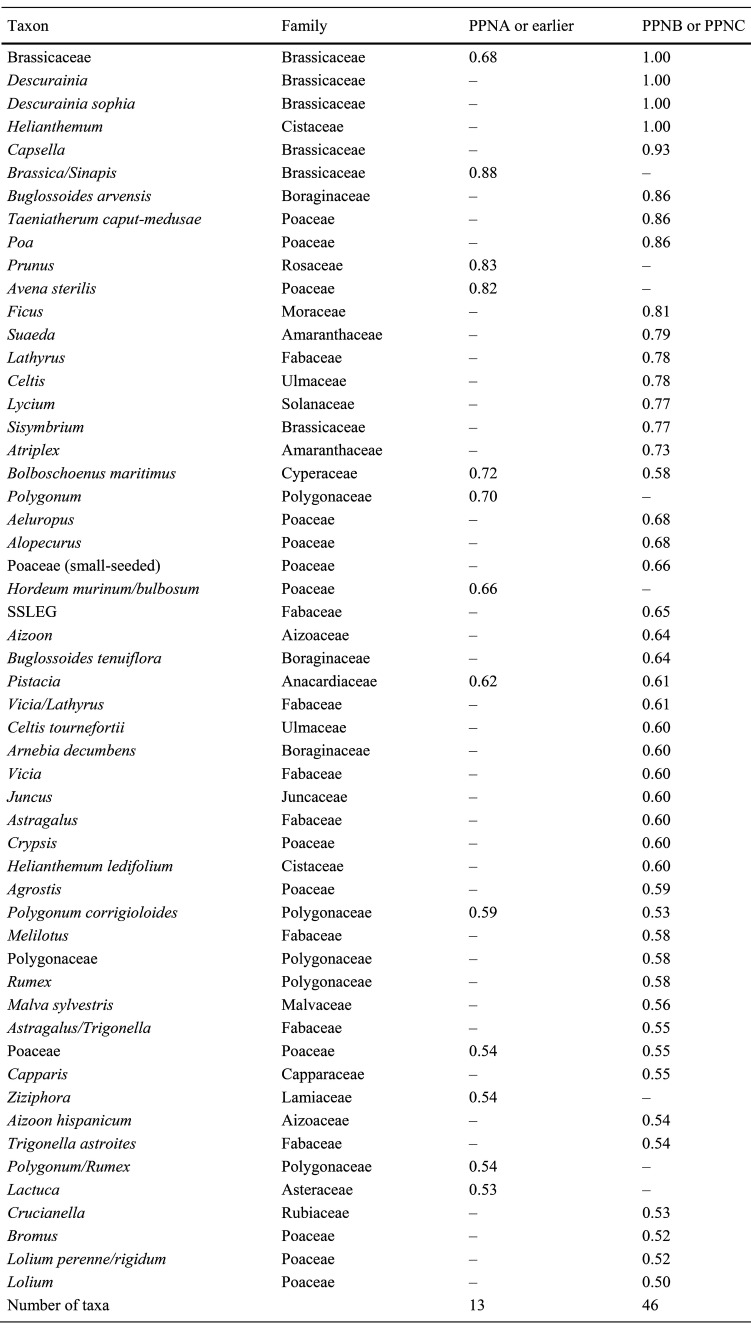
Deliberate collection (DC) scores for wild plant taxa (excluding crop progenitors) with DC scores ≥ 0.5

Alongside the wild species, we considered domesticated crops because these are likely to have been brought onto a site deliberately, and can therefore be used as an independent benchmark for the DC scores likely to indicate a deliberately collected taxon. All of the southwest Asian founder crops except *Cicer arietinum* (chickpea) make up ≥ 30% of at least one sample with a DC score ≥ 0.5, and *Triticum monococcum* (einkorn), *Lens culinaris* (lentil) and *Hordeum vulgare* (barley) have very high DC scores (> 0.9). *Triticum dicoccum* (emmer) derives from a sample with a relatively low score (0.57) but its true representation is probably masked because many glume wheat grains are identified only as *T. monococcum*/*dicoccum*, which makes up ≥ 30% of a sample with a DC score of 0.86. Chickpea, on the other hand, is quite rare overall in the archaeobotanical record for the PPN (Zohary et al. 2012, p 89) and so may not have been widely cultivated. Such rarely found species are relatively unlikely to be represented in stored deposits, which are themselves quite rare.

We also considered wild crop progenitors because these must have been deliberately collected at some stage prior to their domestication. Their representation is complicated, however, by problems of identification (see, for example, Zohary et al. 2012). Wild and domesticated forms of *Lens* spp. (lentil), *Cicer* spp. (chickpea) and *Vicia ervilia* (bitter vetch) are very difficult to distinguish, and distinctions are often based on size alone (or even the date of the deposit). *Lens orientalis* and *Cicer reticulatum*, the wild forms of lentil and chickpea, and bitter vetch are rarely (if ever) recorded as such in archaeobotanical reports. Identifications to genus are therefore very common and include both wild and domesticated forms. The problem of identification is less severe for the cereals, but while the two-grained form of wild einkorn (*T. boeoticum thaoudar*) is often recorded as such, the one-grained form (*T. boeoticum aegilopoides*) is usually included in the more general category “*T. boeoticum*”. Despite these complications, crop progenitors are also well represented in Table 2, by species that can be accurately identified as wild—pea (*P. elatius*) and barley (*H. spontaneum*), and potentially by *Lens* sp., all of which are found in samples with a DC score > 0.8. *T. boeoticum* is also found in a sample with a DC score of 0.45.

Overall, therefore, the representation of domesticated crops and their progenitors suggests that taxa making up greater than 30% of a sample with a DC score ≥ 0.5 provide a reasonable measure by which to assess the likelihood of a taxon being deliberately collected for use. On this basis, about 40 genera of wild plants (excluding crop progenitors) from about 20 families may be considered as potential candidates for deliberate collection (Table 3).

### Distinguishing wild plant foods from plants collected for other uses

Even when a case can be made for the deliberate collection of wild plants, it cannot be assumed that they were collected for food. This must be inferred from the plant’s characteristics (e.g. whether edible, palatable or nutritious), ethnographic evidence and depositional context, such as association with evidence of food preparation or consumption. Here we define wild food plants as those consumed as staples, nutritional supplements or flavourings, and Table 4 gives the recorded uses for food or other purposes of all the wild taxa listed in Table 3 as potential candidates for deliberate collection. The purpose of Table 4 is to assist in distinguishing taxa that are likely to have been collected as food from those more likely to have been collected for other purposes, rather than to differentiate between different types of non-food use.

**Table 4:**
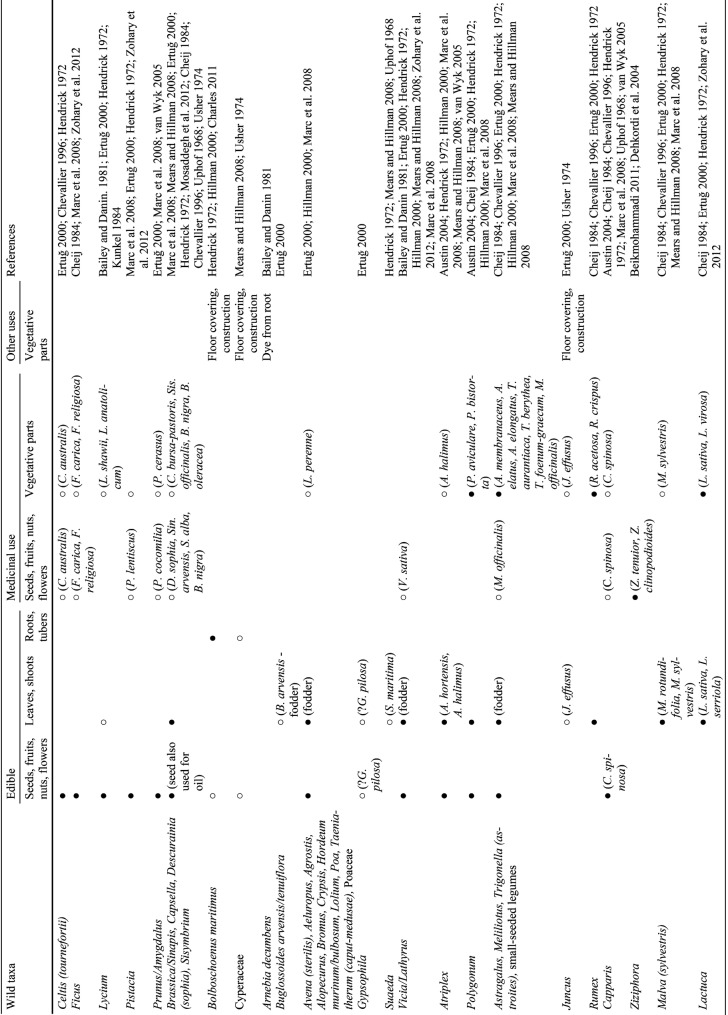
Recorded uses of the wild plant taxa listed in Table 3, taxonomically ordered. NB: no known recorded uses for *Helianthemum *(*ledifolium*), *Aizoon *(*hispanicum*) and *Crucianella*. ^●^➝Widely recognised use. ^○^➝Occasionally/rarely recognised use

Tables 3 and 4 include several fruit and nut taxa (*Pistacia, Ficus, Prunus*/*Amygdalus, Celtis, Lycium*), which may well have been collected as food. The seeds of many of the other wild taxa are also edible, including Poaceae, Fabaceae, Brassicaceae, Cyperaceae, *Atriplex* and *Polygonum*/*Rumex*. Plants collected for their seed have a good chance of being preserved archaeologically, especially if the seeds are parched during processing, as is common for *Polygonum* (Hillman 2000, p 357). The leaves and shoots of several of these taxa are also known ethnographically to be eaten as greens, as are the leaves and shoots of other taxa in Table 4 whose seed is not normally eaten (e.g. *Lactuca, Juncus, Malva sylvestris* and *Suaeda*). The tubers of *Bolboschoenus maritimus*, and other Cyperaceae species such as *Cyperus esculentus*, are another potential source of food (Simpson and Inglis 2001; Dogan et al. 2004; Holec et al. 2014). These tubers are fully formed at about the time when plants are in seed (Jordan-Molero and Stoller 1978; Davis 1986), and seeds may be deposited at settlements if, for example, the above-ground parts were gathered during the uprooting of below-ground organs. Some of the plants in Table 4 can also be used for their oil or as flavouring, (e.g. Brassicaceae and *Capparis*).

As well as food, there is a variety of other ethnographically attested plant uses (Table 4). For example, many plants have been used for their medicinal properties, and those for which their seeds are utilised in this way include *Atriplex, Descurainia sophia* and *Ziziphora*. Reeds and rushes are commonly collected for use as matting, bedding or in construction but, as has been noted for *B. maritimus* and *Juncus* (Hillman 2000), they are often used before they come into seed. Plants could also be used for dyeing (e.g. *Arnebia decumbens*) or decorative purposes (e.g. *Crucianella, Helianthemum* and *M. sylvestris*), and there is one instance of *Helianthemum* seeds stored in a pot within a shrine (at Çatalhöyük) for an unknown, but presumably socially significant, reason.

Although the taxa listed as potential food plants in Tables 3 and 4 have met our criteria for deliberate collection in at least one context, it does not necessarily follow that they arrived on site as foods wherever they were found. There are also incidental routes by which potential food plants may have reached a site as, for example, when fruits or seeds were collected unintentionally with crops, foraged wild species, woody material or animal dung. For example, some mixed samples are very large (those in the upper left of Fig. 2a, b), and it is tempting to conclude that these represent the remains of plant food processing, especially when they derive from hearths or other areas of burning. A closer examination of the botanical composition of such samples, however, suggests an alternative interpretation.

Many of these large, mixed samples are from Neolithic middens at Çatalhöyük, and the plant remains in these deposits are thought to derive partly from discarded household refuse and partly from in situ burning (Bogaard et al. 2013). A comparison of the charred plant remains in samples from one of these middens (Area 181) shows that both fire-spots and midden deposits exhibit a similar range of botanical compositions (see, for example, the adjacent samples D69 and D72 in ESM Fig. D), suggesting that the plant remains derive from the same source. Many of the dominant taxa in these samples grow in saline habitats (*Aeluropus, B. maritimus*) or marshy areas (rushes), and others have small or hard-coated seeds (small-seeded legumes), Chenopodiaceae, Brassicaceae, *Helianthemum* (*ledifolium*) that survive passage through the ruminant digestive system (Miller and Smart 1984; Charles 1996; Wallace and Charles 2013). These, and several less dominant small-seeded taxa (*Sporobolus* (saline habitats), *Chenopodium chenopodioides, Juncus* (marshy areas), *Alopecurus, Artemisia annua*), are suggestive of grazing habitats, and the burning of dung fuel has been suggested as a major contributor of the plant remains found in these deposits (Bogaard et al. 2013; Filipović 2014). The Çatalhöyük samples are not unique in this respect; for example, Epipalaeolithic samples from Abu Hureyra, including a large mixed sample, which plots with the large Çatalhöyük midden samples, are also composed of taxa consistent with derivation from dung (Miller 1996).

So, even taxa that were sometimes brought to site as foods could, at other times and places, have arrived on site by other routes. These include taxa likely to survive ruminant digestion (e.g. Cyperaceae—especially *B. maritimus—Juncus, Atriplex, Polygonum, Rumex* and small-seeded legumes) that are as likely to have been deposited in dung fuel as collected for food, especially with the development of animal management (Matthews 2016). Other plants, including some of the same taxa, may also have been collected for purposes other than food, such as for building materials (Cyperaceae, especially *B. maritimus* and *Juncus*) or dye plants (*Arnebia decumbens*). The use of these taxa as food cannot therefore be universally inferred.

### The chronological and geographical distribution of deliberately collected wild food plants

We compared the wild plant foods recognised in pre-domestication periods with those following the advent of domesticated crops in order to evaluate the evidence for a narrowing of the wild plant food spectrum during the transition to agriculture. For samples dated to the PPNA or earlier, and so before the emergence of domesticated crops, several taxa were identified that best meet our criteria for recognition as wild plant foods, based on their composition—(Table 3) and known use as food—(Table 4). These include wild grasses (Poaceae, especially *Avena sterilis* and *Hordeum bulbosum*/*murinum*), fruits and nuts (*Prunus*/*Amygdalus, Pistacia* and *Ficus*), members of the Brassicaceae (especially *Brassica*/*Sinapis*) and other herbaceous species (*B. maritimus, Lactuca*, and *Polygonum*/*Rumex* species), of which *Lactuca* and *Rumex* are primarily eaten as greens and are less likely to be represented by their seeds. Of these, only *Avena sterilis, Prunus*/*Amygdalus, Brassica*/*Sinapis, B. maritimus* and *Polygonum* came from a sample with a DC score of ≥ 0.7 and so from the area of the “abundance-purity” plot associated with storage contexts (Fig. 3a, b). *B. maritimus* and *Polygonum*/*Rumex* may also have been deposited on some occasions as a result of the burning of dung, whether from wild or managed animals. Though it has been argued that the collection of dung from wild animals is unlikely (Hillman et al. 1989, 1997; Savard et al. 2006), ethnographic evidence and counter-arguments have been presented to support the collection wild animal dung as a source of fuel (Miller 1996, 1997).

Using the same criteria, a greater number of wild taxa were recognised as potentially collected in later PPN periods (Early PPNB to PPNC), after the emergence of domesticated crops. These include *Descurainia (sophia), Capsella* and *Sisymbrium* species (and perhaps other members of the Brassicaceae), fruits and nuts (*Ficus, Pistacia* and *Celtis (tournefortii)*), wild grasses (including *Taeniatherum caput-medusae, Poa, Aeluropus, Alopecurus, Crypsis, Agrostis, Bromus* and *Lolium* species), wild legumes (Fabaceae), both large-seeded (*Vicia*/*Lathyrus*) and small-seeded (including *Trigonella (astroites*), *Astragalus*, and *Melilotus* species), and a wide range of other herbaceous species, including members of the Boraginaceae (*Buglossoides arvensis, B. tenuiflora* and *Arnebia decumbens*) (Table 3). Of these, only the Brassicaceae and Boraginaceae species, *Ficus, Celtis, Taeniatherum caput-medusae, Poa, Lathyrus* and the herbaceous species, *Helianthemum, B. maritimus, Atriplex, Suaeda* and *Lycium* came from a sample with a DC score of ≥ 0.7, suggestive of storage (Fig. 3a, b). The use of dung fuel is also well documented in this period (Matthews 2016), which may account for some of the occurrences of taxa such as *Helianthemum, B. maritimus, Atriplex* and *Suaeda*.

Few taxa were recognised as likely to have been deliberately collected in more than one of the five geographical regions in Fig. 1 (central Anatolia, Cyprus, southern and northern Levant, and the eastern Fertile Crescent). Those that do occur widely tend to be taxonomically diverse groupings, such as small-seeded legumes, Brassicaceae and small-seeded grasses (Table 3). The exceptions to this are *B. maritimus, Polygonum* and/or *Rumex*, which are found in multiple regions (ESM Table D). Moreover, within each region it is unusual for a taxon to be recognised as deliberately collected at more than one or two sites. Indeed, only 12 wild taxa excluding crop progenitors were found at more than one site, and many of these are those likely to be over-represented due to preservation bias—such as Boraginaceae species, the nutlets of which are encased in a siliceous outer coat, or fruits represented by their robust waste product (fruit stones). Rather than indicating genuine regional patterns in plant use, this probably reflects the difficulty of finding reliable evidence for deliberate collection.

## Discussion

### The recognition of deliberately collected food plants

When recognising wild plant collection, there are two aspects that need to be considered: first, what constitutes strong evidence for deliberate collection and secondly, how reliably can we recognise this evidence? Both storage for future use and processing prior to use can be considered strong evidence of collection. However, for pre-agricultural communities exploiting (and possibly cultivating) a range of wild plants available in different seasons, storage may have been a less essential (and, if these communities were also mobile, even impractical) element of plant usage than it is for agricultural communities based on grain crops available in one season only. At the Upper Palaeolithic site of Ohalo II (Weiss et al. 2008) and the EPPNB site of Tell Qarassa (Arranz-Otaegui et al. 2016), for example, there is contextual evidence for wild plant food processing but not for storage. Evidence for feasting in the form of large-scale food processing and cooking also increases through the PPN period (Twiss 2008), which provides greater opportunity for plant material to be preserved through charring. Moreover, taxa that are primarily represented by their waste products, such as fruit stones and nutshell, are more likely to be found in processing than storage contexts. For this reason, our approach has focused on recognising both the storage and processing of wild plant material in the archaeological record.

With regards to reliability, however, while contextual evidence of plant processing is occasionally found, as at Ohalo II (see above; Weiss et al. 2008), the most unambiguous evidence for deliberate collection of a wild plant is usually evidence of its storage. Our comparison of contextual evidence and sample composition across southwest Asia has indicated that, where good contextual information is available, it is only likely storage contexts (such as containers and burnt destruction deposits) that are associated with archaeobotanical samples that are both rich and pure; other known archaeological contexts (such as refuse deposits and external burnt areas) have not produced such samples. Thus compositional evidence alone (a high DC score indicating a combination of seed abundance and sample purity) provides a reliable indication of deliberate collection, usually in the form of storage. On the other hand, samples with intermediate DC scores provide more ambiguous evidence of deliberate collection, as they may derive from a number of different sources, including plant processing and mixed storage, but also refuse or animal dung. This ambiguity also applies to the large but mixed samples commonly recovered from refuse deposits, where such large quantities of charred remains are more likely to result from the deliberate burning of dung fuel than the accidental burning of food processing remnants (Miller and Smart 1984; Miller 1984). Many, if not most, of the taxa from these samples may therefore derive from the use of dung fuel.

So, while both storage and processing constitute equally valid evidence for plant collection, storage can be more reliably indicated from compositional evidence (purity and density) alone whilst the recognition of processing must remain uncertain unless backed up by strong contextual evidence such as their association with ground stone tools. Such evidence can be difficult to find, however, so while it is possible to recognise taxa that *were* deliberately collected it is more difficult to say which taxa were *not*.

### The broad spectrum of plant food collection?

We have brought together the botanical composition of samples, their archaeological context and the potential use of the taxa dominant in samples, to systematically recognise those wild plants for which a good case can be made for their deliberate collection as food. On this basis, relatively few of the wild taxa found at pre- and early agricultural sites can be confidently recognised as contributing to the human diet at one time or another during the PPN, though we acknowledge that this group represents the *minimum* number of wild taxa used as food. In addition, some foods, such as fruits and nuts, may be overlooked because they are usually represented by the waste by-products of consumption, such as seeds and shell, rather than by the product consumed.

We can now re-evaluate the spectrum of wild food plants at pre-agricultural and early agricultural sites. One of the largest groups of wild taxa represented on pre-agricultural (PPNA and earlier) sites is the grasses, and these are usually assumed to have been collected for food. Our evidence indicates, however, that of the 40–50 genera of grasses represented in southwest Asia, there is strong evidence for the deliberate collection of only ten genera, each of which may represent no more than one collected species. On the other hand, our evidence has provided support for the deliberate collection of some taxa that have previously been only tentatively suggested as plant foods on the grounds of their overall abundance or ethnographic use, for example *B. maritimus* (Savard et al. 2006, p 189), *Atriplex* (de Moulins 1997, p 93) and *Polygonum* (Hillman 2000, pp 357–358).

To some extent, therefore, our results have called into question the exploitation of a wide range of plant species by pre-agricultural communities in southwest Asia in the period leading up to domestication. Moreover, comparing the evidence from pre-agricultural sites, for which few wild food plants have been confidently recognised, with that from later proto- and early agricultural sites (EPPNB to PPNC), there is evidence for the collection of a broader range of wild plant foods at the latter, though this may reflect the greater quantity of evidence and the more frequent occurrence of unambiguous storage contexts and cooking or other processing involving fire. For these reasons, while we do not interpret this contrast as indicating an increase in the diversity of wild plant foods collected at these later PPN sites, we have found no evidence for a narrowing of the plant food spectrum during the adoption of agriculture.

Rather, our results, based on taxa for which there is reliable evidence of their deliberate collection as food, suggest little change in the variety of wild plant foods exploited between pre-agricultural and proto-/early agricultural periods, despite the availability of potentially higher ranked domesticated crops in the later period. This implies a persistence in opportunistic foraging throughout the PPN, and, if it occurred in resource-rich environments and encouraged increased sedentism, would have provided ample opportunity for experimentation in new exploitation techniques, during which time the cultivation of a range of different species could be trialled (Smith 2007, 2011b, 2016; Zeder 2012, 2015, 2016).

An important consequence of this interpretation of the archaeobotanical evidence is that it does not require an explanation for the emergence of agriculture to be based on an externally-driven demographic or environmental “push” model, whereby foragers were forced to exploit lower ranked plant species (in response to resource depletion) that subsequently declined with the advent of domesticated crops. Rather, continued opportunistic foraging may have provided a context for the development of a mutualistic relationship between people and certain plants, with both taking advantage of favourable conditions, a diverse and plentiful resource base for people, and a rich anthropogenic environment for plants—that is to the evolutionary benefit of both and led ultimately to crop domestication and a dependence on agriculture (Rindos 1980, 1984; Smith 2007, 2011b; Zeder 2015, 2016).

## Conclusions

For the first time, the botanical composition of individual archaeobotanical samples from across southwest Asia has been systematically combined with their archaeological context, with the aim of establishing a robust link between archaeological evidence and its interpretation in terms of deliberate collection and use of plants as food. This has put the recognition of wild plant foods on a firm footing, and has led to the recognition of a suite of wild plant taxa for which there is strong evidence for their exploitation as plant foods at pre-agricultural and early agricultural sites in southwest Asia. This has shown that relatively few of the wild taxa found at pre- and early agricultural sites can be confidently recognised as contributing to the human diet. The approach adopted here has resulted in a re-evaluation of the evidence for the exploitation of a broad spectrum of wild plant foods at pre-agricultural sites, and its supposed narrowing during the early development of agriculture. This has implications for how we understand the processes leading to the domestication of crops, and points towards a mutualistic relationship between people and plants as a driving force during the development of agriculture.

## Electronic supplementary material

Below is the link to the electronic supplementary material.

Supplementary material 1 (DOC 922 KB)
