# Supplementary material for: Re-analysis of archaeobotanical remains from pre- and early agricultural sites provides no evidence for a narrowing of the wild plant food spectrum during the origins of agriculture in southwest Asia
Source: Veg Hist Archaeobot. 2018 Nov 17;28(4):449–63. doi: 10.1007/s00334-018-0702-y (PMC6551342; doi:10.1007/s00334-018-0702-y)
Supplement: Supplementary file 1 — Supplementary material 1 (DOC 922 KB) [file 334_2018_702_MOESM1_ESM.doc]

**Supplementary Figure A**

The Simpson and Shannon indices for synthetic samples composed of varying proportions of taxa (each colour representing a different taxon, with orange indicating the dominant taxon in cases where one taxon predominates). Note that, for example, samples D-G have very similar Simpson indices but very different Shannon indices. Since we are primarily interested in the taxa that make up a large proportion of a sample the Simpson index, therefore, best represents the purity of the sample.


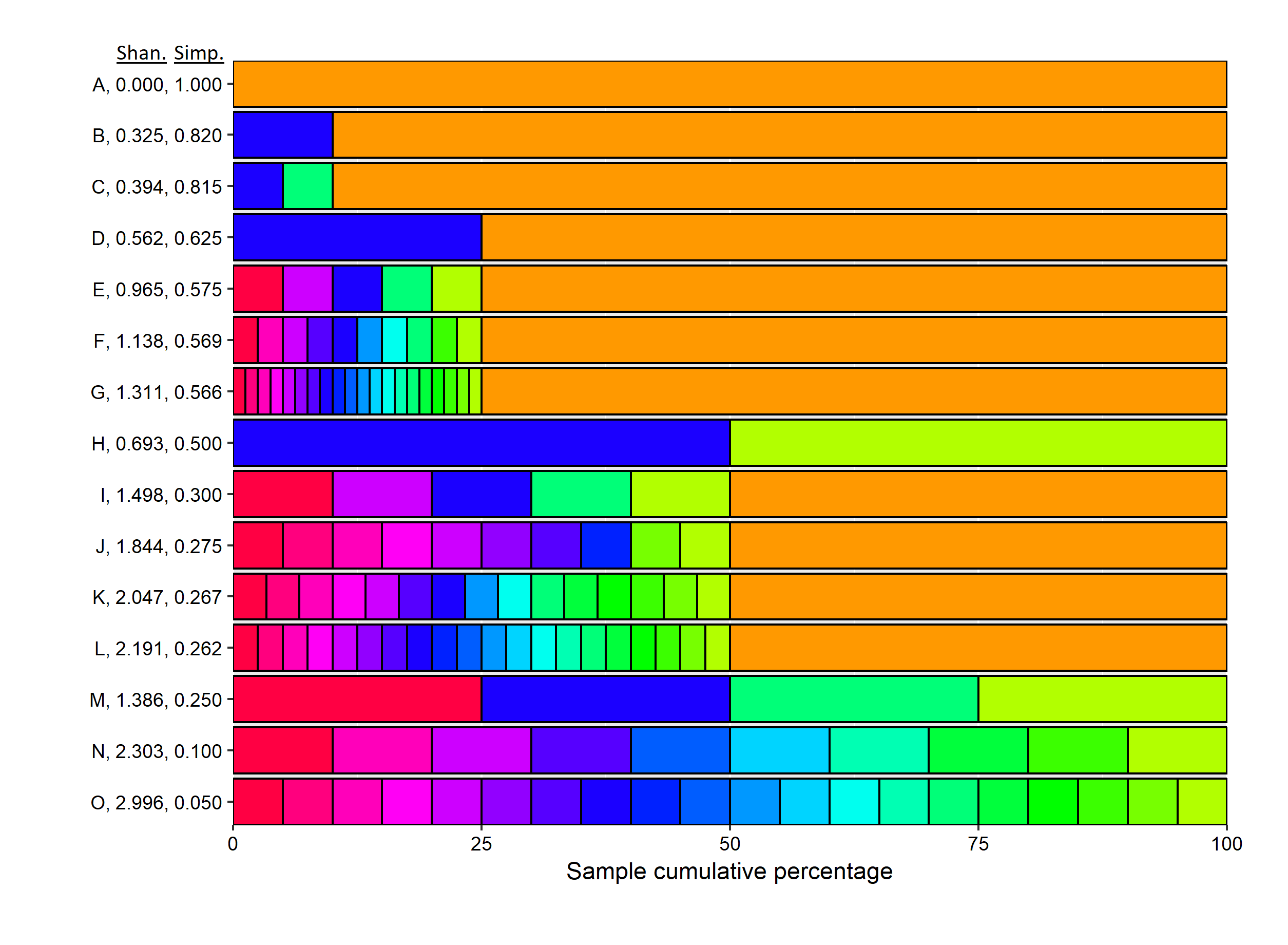


**Supplementary Figure B**

Histogram of total number of plant items (on a logarithmic scale) in archaeobotanical samples included in the database.


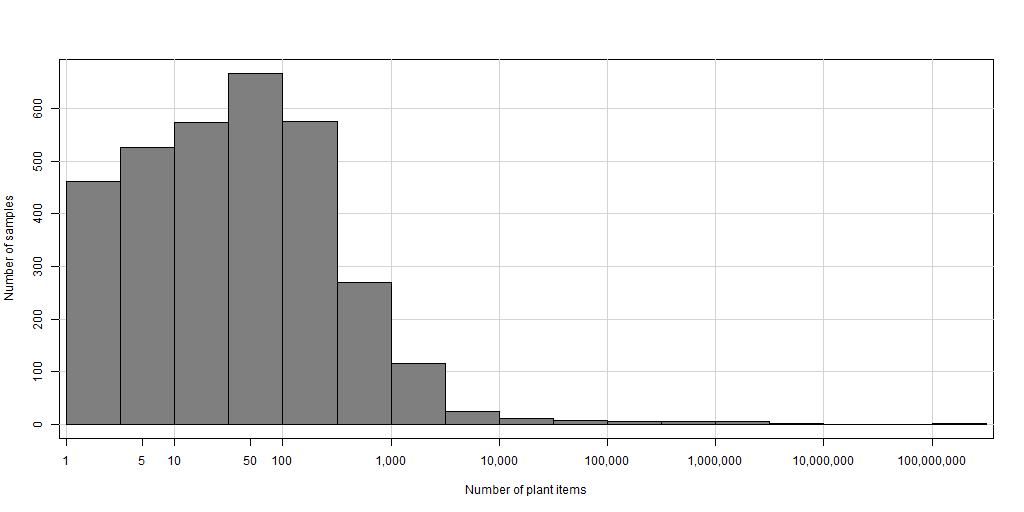


**Supplementary Figure C**

Counts of plant items in archaeobotanical samples plotted (on a logarithmic scale) against Simpson index (small and mixed samples – see Supplementary Information, Equation A – not plotted). Red (filled) symbols indicate samples from the following context categories: (a) internal spaces, (b) external spaces. Contour lines indicate density estimations for each context category: multiple lines indicate increasing concentrations of samples.


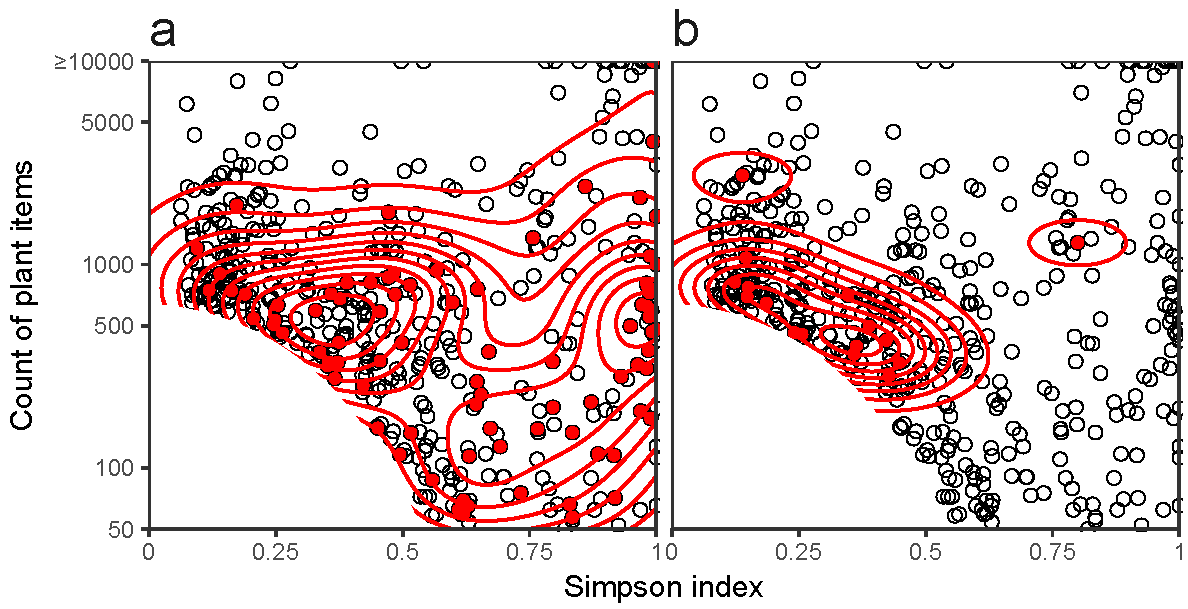


**Supplementary Figure D**

Pie charts showing the botanical composition of samples from a midden (Area 181) at Çatalhöyük. Taxa represented in the midden by fewer than 300 plant items are excluded. Family categories include members of the family not independently reaching 300 items. Key indicates the most commonly occurring wild taxa, in descending order of their total count within the midden. The white segment indicates cereals (mostly domesticated).


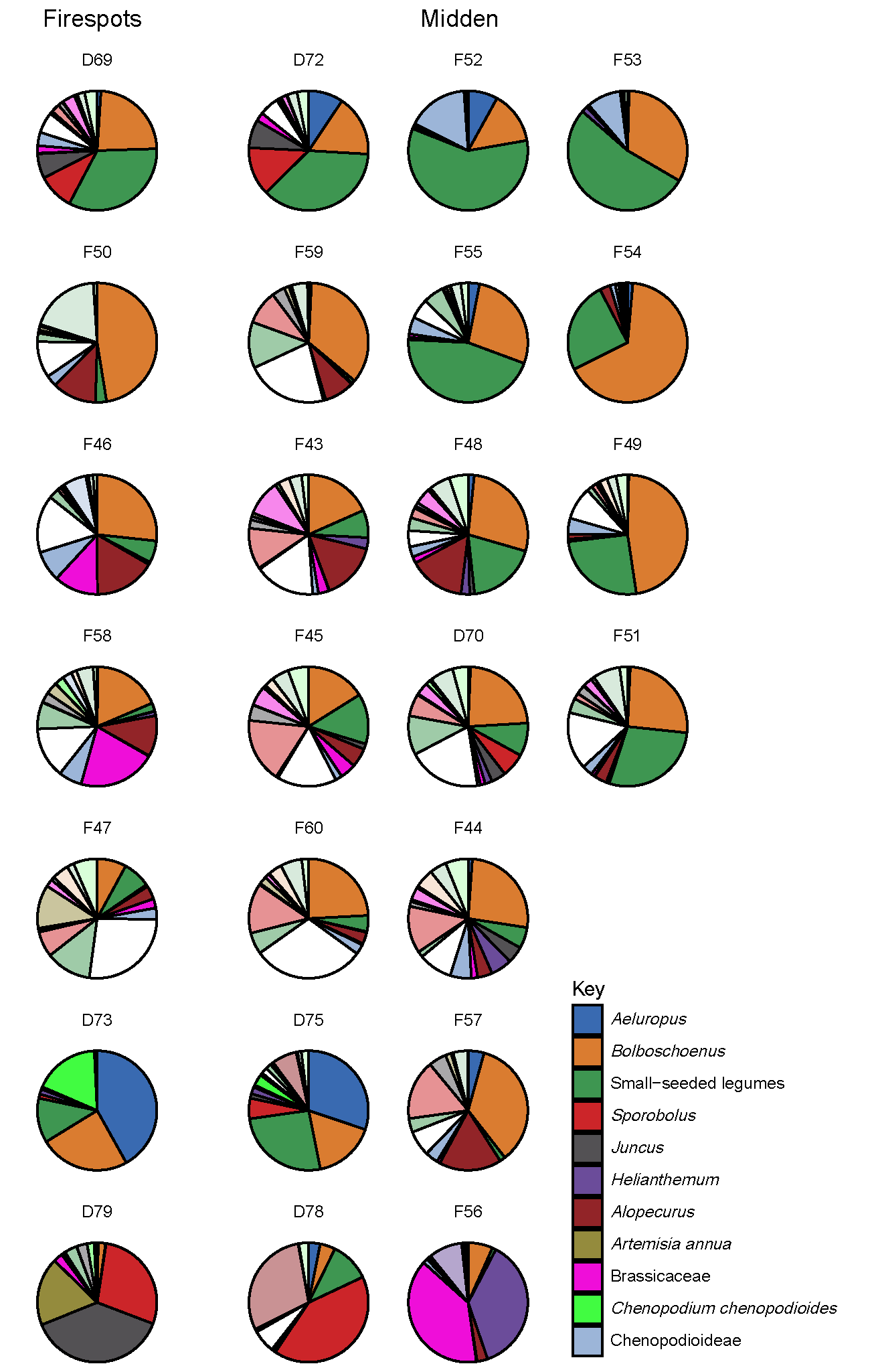


**Supplementary Table A**

Number of published and unpublished samples for each site used in the analysis, and the original data sources.

| **Site** | **Published** | **Unpublished** | **Sources** |
| --- | --- | --- | --- |
| Abu Hureyra | 114 | 0 | de Moulins 1997; Moore et al. 2000 |
| Ais Yiorkis | 24 | 0 | Lucas et al. 2012 |
| Aşikli Höyük | 176 | 0 | van Zeist and de Roller 1995; van Zeist and de Roller 2003b |
| Azraq 31 | 29 | 0 | Colledge 2001 |
| Basta I | 92 | 0 | Neef 2004 |
| Beidha | 9 | 0 | Colledge 2001; Helbæk 1966 |
| Cafer Höyük | 62 | 0 | de Moulins 1997 |
| Can Hasan III | 4 | 0 | French et al. 1972 |
| Çatalhöyük East | 312 | 159 | Fairbairn 2007; Fairbairn et al. 2002; Filipović 2012; Bogaard pers. comm.; Fairbairn, pers. comm. |
| Çayönü | 245 | 0 | van Zeist and de Roller 1994; van Zeist and de Roller 2003a |
| Chogha Bonut | 24 | 0 | Miller 2003 |
| Chogha Golan | 3 | 0 | Riehl et al. 2012, 2013 |
| Chia Sabz | 1 | 3 | Riehl et al. 2012; Riehl, pers. comm. |
| Demirkoy | 0 | 12 | Savard 2004; Savard et al. 2006 |
| Dhuweilla | 7 | 0 | Colledge 2001 |
| Dj'ade | 0 | 219 | Willcox 1996; Willcox et al. 2008; Willcox; pers. comm. |
| el-Hemmeh | 0 | 45 | White 2013; White and Makarewicz 2012; White, pers. comm. |
| El Kowm I | 8 | 0 | van Zeist 1986 |
| El Kowm II | 31 | 0 | de Moulins 1997 |
| Ganj Dareh Tepe | 122 | 0 | van Zeist et al. 1984 |
| Gilgal | 1 | 0 | Weiss et al. 2006 |
| Gritille | 0 | 52 | Miller 2013 |
| Hallan Çemi | 0 | 175 | Rosenberg et al. 1998; Savard 2004 |
| Hacılar | 9 | 0 | Helbæk 1970 |
| Iraq ed-Dubb | 13 | 0 | Colledge 2001 |
| Jerf el Ahmar | 1 | 258 | Willcox 1996; 2002; Willcox and Fornite 1999; Willcox et al. 2008; Willcox, pers. comm. |
| Jericho | 18 | 0 | Hopf 1983 |
| Kastros | 23 | 0 | van Zeist 1981 |
| Mlefaat | 4 | 0 | Savard et al. 2003 |
| Mureybet | 65 | 0 | van Zeist and Bakker-Heeres 1986b |
| Mylouthkia | 12 | 0 | Murray 2003 |
| Nevali Çori | 0 | 237 | Pasternak 1998; Pasternak, pers. comm. |
| Qermez Dere | 0 | 45 | Nesbitt 1995; Savard 2004; Watkins et al. 1991 |
| Sheikh-e Abad | 0 | 40 | Whitlam 2015 |
| Tell Abr | 0 | 29 | Willcox et al. 2008; Willcox; pers. comm. |
| Tell Aswad | 30 | 0 | van Zeist and Bakker-Heeres 1985 |
| Tell Bouqras | 28 | 0 | van Zeist and Waterbolk-van Rooijen 1985 |
| Tell Ghoraifé | 35 | 0 | van Zeist and Bakker-Heeres 1985 |
| Tell Maghzaliyeh | 3 | 0 | Willcox n.d. |
| Tell Qaramel | 0 | 108 | Willcox et al. 2008; Willcox, pers. comm. |
| Tell Ramad | 47 | 0 | van Zeist and Bakker-Heeres 1985 |
| Tell Ras Shamra | 62 | 0 | van Zeist and Bakker-Heeres 1986a |
| Tell Sabi Abyad II | 15 | 0 | van Zeist and de Rommer 2000 |
| Wadi Faynan 16 | 43 | 0 | Kennedy 2007 |
| Wadi Fidan A | 4 | 0 | Colledge 2001 |
| Wadi Fidan C | 5 | 0 | Colledge 2001 |
| Wadi al-Hammeh 27 | 14 | 0 | Colledge 2001 |
| Wadi el-Jilat 6 | 10 | 0 | Colledge 2001 |
| Wadi el-Jilat 7 | 25 | 0 | Colledge 2001 |
| Wadi el-Jilat 13 | 28 | 0 | Colledge 2001 |
| Yarym Tepe | 12 | 0 | Bakhteyev and Yanushevich 1980 |
| Zahrat adh-Dhra 2 | 10 | 0 | Edwards et al. 2004 |

**Bibliography for Supplementary Table A**

Bakhteyev FK, Yanushevich ZV (1980) Discoveries of cultivated plants in the early farming settlements of Yarym-Tepe I and Yarym-Tepe II in Northern Iraq. Journal of Archaeological Science 7:167-178

Colledge S (2001) Plant exploitation on Epipalaeolithic and early Neolithic sites in the Levant. (British Archaeological Reports International Series 986), Archaeopress, Oxford

de Moulins D (1997) Agricultural changes at Euphrates and steppe sites in the mid-8th to the 6th millennium B.C. (British Archaeological Reports International Series 683), Archaeopress, Oxford

Edwards PC, Meadows J, Sayej G, Westaway M (2004) From the PPNA to the PPNB: new views from the southern Levant after excavations at Zahrat adh-Dhra' 2 in Jordan. Paléorient 30:21-60

Fairbairn A (2007) Wild plant seed storage at Neolithic Çatalhöyük East, Turkey. Vegetation History and Archaeobotany 16:467-479

Fairbairn A, Asouti E, Near J, Martinoll D (2002) Macro-botanical evidence for plant use at Neolithic Çatalhöyük, south-central Anatolia, Turkey. Vegetation History and Archaeobotany 11:41-54

Filipović D (2012) An archaeobotanical investigation of plant use, crop husbandry and animal diet at early-mid Neolithic Çatalhöyük, Central Anatolia Doctoral thesis, University of Oxford

French D, Hillman G, Payne S, Payne R (1972) Excavations at Can Hasan III 1969-1970. In: Higgs E (ed) Papers in economic prehistory. Cambridge University Press, London, pp 181-190

Helbæk H (1966) Pre-Pottery Neolithic farming at Beidha. Palestine Exploration Quarterly 98:61-66

Helbæk H (1970) The plant husbandry of Hacılar. In: Mellaart J (ed) Excavations at Hacilar. Edinburgh University Press, Edinburgh, pp 189-244

Hopf M (1983) Jericho plant remains. In: Kenyon KM, Holland TA (eds) Excavations at Jericho V: the Pottery phases of the tell and other finds. British School of Archaeology in Jerusalem, London

Kennedy A (2007) The plant macrofossils. In: Finlayson B, Mithen S (eds) The early prehistory of Wadi Faynan, southern Jordan: Archaeological survey of wadis Faynan, Ghuwayr and al-Bustan and evaluation of the Pre-Pottery Neolithic A site of WF16. Oxbow Books, Oxford

Lucas L, Colledge S, Simmons A, Fuller DQ (2012) Crop introduction and accelerated island evolution: archaeobotanical evidence from ‘Ais Yiorkis and Pre-Pottery Neolithic Cyprus. Vegetation History and Archaeobotany 21:117-129

Miller N (2003) Plant remains from the 1996 excavation. In: Alizadeh A (ed) Excavations at the prehistoric mound of Chogha Bonut, Khuzestan, Iran. The University of Chicago, Chicago, pp 123-128

Miller N (2013) Plant remains from Neolithic Gritille: food and fuel in the context of animal domestication. MASCA Ethnobotanical Laboratory Report 27:1-21

Moore A, Hillman G, Legge A (2000) Village on the Euphrates. From foraging to farming at Abu Hureyra. Oxford University Press, New York

Murray MA (2003) The plant remains. In: Peltenberg E (ed) The colonisation and settlement of Cyprus. Paul Åströms Förlang, Sävedalen, pp 59-71

Neef R (2004) Vegetation and climate. A comparison between PPNB 'Ain Ghazal and Basta. In: Bienert H-D, Gebel H, Neef R (eds) Central settlements in Neolithic Jordan. (Studies in early Near Eastern production, subsistence and environment 5) ex Oriente, Berlin

Nesbitt M (1995) The search for plant remains. In: Watkins T (ed) Qermez Dere, Tell Afar: interim report No 3. Department of Archaeology, University of Edinburgh, Edinburgh, pp 9-45

Pasternak R (1998) Investigations of botanical remains from Nevali Çori PPNB, Turkey: a short interim report. In: Damania AB, Valkoun J, Willcox G, Qualset CO (eds) The origins of agriculture and crop domestication. ICARDA, Aleppo

Riehl S, Benz M, Conard NJ, Darabi H, Deckers K, Nashli HF, Zeidi-Kulehparcheh M (2012) Plant use in three Pre-Pottery Neolithic sites of the northern and eastern Fertile Crescent: a preliminary report. Vegetation History and Archaeobotany 21:95-106

Riehl S, Zeidi M, Conard NJ (2013) Emergence of agriculture in the foothills of the Zagros Mountains of Iran. Science 341:65

Rosenberg M, Nesbitt RM, Redding RW, Peasnall BL (1998) Hallan Çemi, pig husbandry, and post-Pleistocene adaptations along the Taurus-Zagros Arc (Turkey). Palaeohistoria 24:25-41

Savard M (2004) Epipalaeolithic to early Neolithic subsistence strategies in the northern Fertile Crescent. Doctoral thesis, University of Cambridge

Savard M, Nesbitt M, Gale R (2003) Archaeobotanical evidence for early Neolithic diet and subsistence at M'lefaat (Iraq). Paléorient 29:93-106

Savard M, Nesbitt M, Jones MK (2006) The role of wild grasses in subsistence and sedentism: new evidence from the northern Fertile Crescent. World Archaeology 38:179-196

Van Zeist W (1981) Plant remains from Cape Andreas - Kastros (Cyprus). In: le Brun A (ed) Un site néolithique précéramique en Chypre: Cap Andreas-Kastros. (Recherche sur les grandes civilisations, vol 5) Editions ADPF, Paris, pp 95-99

Van Zeist W (1986) Plant remains from Neolithic El Kowm, central Syria. In: Dornemann RH (ed) A Neolithic village at Tell El Kowm in the Syrian desert. (Studies in ancient oriental civilization, vol 43) University Chicago, Oriental Institute, Chicago, pp 65-68

Van Zeist W, Bakker-Heeres J (1985) Archaeobotanical studies in the Levant 1. Neolithic sites in the Damascus basin: Aswad, Ghoraifé, Ramad. Palaeohistoria 24:165-256

Van Zeist W, Bakker-Heeres J (1986a) Archaeobotanical studies in the Levant 2. Neolithic and Halaf levels at Ras Shamra. Palaeohistoria 26:151-170

Van Zeist W, Bakker-Heeres J (1986b) Archaeobotanical studies in the Levant 3. Late-Palaeolithic Mureybit. Palaeohistoria 26:171-199

Van Zeist W, de Roller G (1994) The plant husbandry of Aceramic Çayönü, SE Turkey. Palaeohistoria 33/34:65-96

Van Zeist W, de Roller GJ (1995) Plant remains from Asikli Höyük, a pre-pottery Neolithic site in central Anatolia. Vegetation History and Archaeobotany 4:179-185

Van Zeist W, de Roller GJ (2003a) The Çayönü archaeobotanical record. In: van Zeist W (ed) Reports on archaeobotanical studies in the Old World. Groningen, pp 143-166

Van Zeist W, de Roller GJ (2003b) Some notes on the plant husbandry of Aşikli Höyük. In: van Zeist W (ed) Reports on archaeobotanical studies in the Old World. Groningen, pp 115-142

Van Zeist W, de Rommer G (2000) The plant remains. In: Verhoeven M, Akkermans P (eds) Tell Sabi Abyad II the Pre-Pottery Neolithic B settlement. Nederlands Historisch-Archaeologisch Instituut, Istanbul, pp 137-146

Van Zeist W, Waterbolk-van Rooijen W (1985) The palaeobotany of Tell Bouqras, eastern Syria. Paléorient 11:131-147

Van Zeist W, Smith PEL, Palfenjer-Vegter RM, Suwijn M, Casparie WA (1984) An archaeobotanical study of Ganj Dareh Tepe, Iran. Palaeohistoria 26:201-224

Watkins T, Betts A, Dobney K, Nesbitt M (1991) Qermez Dere, Tel Afar: interim report no 2. Department of Archaeology, University of Edinburgh, Edinburgh.

Weiss E, Kislev ME, Hartmann A (2006) Autonomous cultivation before domestication. Science 312:1608-1610

White C (2013) The emergence and intensification of cultivation practices at the Pre-Pottery Neolithic site of el-Hemmeh, Jordan: an archaeobotanical study. Doctoral thesis, Boston University

White CE, Makarewicz CA (2012) Harvesting practices and early Neolithic barley cultivation at el-Hemmeh, Jordan. Vegetation History and Archaeobotany 21:85-94

Whitlam J (2015) Plant use and Neolithic societies of the eastern Fertile Crescent c. 10,000 - 5500 BC. University of Oxford

Willcox G (1996) Evidence for plant exploitation and vegetation history from three Early Neolithic pre-pottery sites on the Euphrates (Syria). Vegetation History and Archaeobotany 5:142-152

Willcox G (2002) Charred plant remains from a 10th millennium B.P. kitchen at Jerf el Ahmar (Syria). Vegetation History and Archaeobotany 11:55-60

Willcox G (n.d.) Magzalia 1979. http://g.willcox.pagesperso-orange.fr/magzaliaseeds.htm. Accessed 15/05/2015 2015

Willcox G, Fornite S (1999) Impressions of wild cereal chaff in pisé from the 10th millennium uncal B.P. at Jerf et Ahmar and Mureybet: Northern Syria. Vegetation History and Archaeobotany 8:21-24

Willcox G, Fornite S, Herveux L (2008) Early Holocene cultivation before domestication in northern Syria. Vegetation History and Archaeobotany 17:313-3

**Supplementary Table B**

Number of samples assigned to each context category per period.

| **Context category** | **PPNA and earlier** | **PPNB/C** |
| --- | --- | --- |
| Internal hearth or oven | 2 | 16 |
| External firespot | 3 | 38 |
| Burnt destruction deposit | 0 | 74 |
| Internal | 27 | 66 |
| External | 9 | 7 |
| Midden | 4 | 36 |
| Pit | 8 | 17 |
| Vessel | 0 | 17 |
| unknown | 35 | 118 |

**Supplementary Table C**

The quantitative archaeobotanical dataset for Ohalo II is unpublished, but the total counts for the 13 taxa that represent 80% of the plant assemblage from Floor II of Hut 1 are listed by Weiss et al. (2008, p 2402, Table 1), and the percentages of (at least some of) these taxa that are found in square F78c are also given (Weiss et al. 2008, p 2404). Based on these figures, it is possible to calculate the numbers of these taxa in F78c.

| Taxon | Total number across Floor II | %age found in square F78c | Number found in square F78c |
| --- | --- | --- | --- |
| *Bromus pseudobrachystachys/tigridis* | 9904 | 15 | 1486 |
| *Hordeum Marinum/hystrix* | 505 | 45 | 227 |
| *H. spontaneum* | 606 | 16 | 97 |
| *Pipatherum holciforme* | 819 | 36 | 295 |
| *Silybum marianum* | 115 | 13 | 15 |
| *Malva parviflora* | 594 | 24 | 143 |
| *Melilotus indicus* | 134 | 58 | 78 |
| *Fumaria macrocarpa* | 31 | 85 | 26 |
| *Aegilops* spp. | 55 | 60 | 33 |

Although not all of the taxa found in F78c are listed above, the implication is that these taxa are the main components of the plant assemblage from this square. The total assemblage would be somewhat larger and more mixed, such that its true position in Figure 2b would be above and to the left of that indicated by the asterisk.

**Supplementary Table D**

List of taxa that comprise ≥30% of the seed count in a sample with a DC score ≥0.5, and details of the samples (site, sample identifier, region, context category and period).

| **Taxon** | **Family** | **DC** | **Site** | **Region** | **Period** | **Context** |
| --- | --- | --- | --- | --- | --- | --- |
| Unidentified | | 0.830444 | Wadi al-Hammeh 27 | S Levant | Pre/PPNA | Internal |
| Unidentified | | 0.804039 | Wadi al-Hammeh 27 | S Levant | Pre/PPNA | Internal |
| Unidentified | | 0.7983 | Wadi al-Hammeh 27 | S Levant | Pre/PPNA | Internal |
| Unidentified | | 0.732461 | Wadi al-Hammeh 27 | S Levant | Pre/PPNA | Internal |
| Unidentified | | 0.694925 | Wadi al-Hammeh 27 | S Levant | Pre/PPNA | Internal |
| Unidentified | | 0.689306 | Wadi al-Hammeh 27 | S Levant | Pre/PPNA | Internal |
| Unidentified | | 0.666165 | Iraq ed-Dubb | S Levant | Pre/PPNA | Internal |
| Unidentified | | 0.615781 | Wadi al-Hammeh 27 | S Levant | Pre/PPNA | Internal |
| Unidentified | | 0.383199 | Dj'ade | N Levant | PPNB/C | Midden |
| Aizoon | Aizoaceae | 0.644478 | Wadi el-Jilat 13 | S Levant | PPNB/C | unknown |
| Aizoon | Aizoaceae | 0.621001 | Wadi el-Jilat 13 | S Levant | PPNB/C | unknown |
| Aizoon | Aizoaceae | 0.5384 | Wadi el-Jilat 13 | S Levant | PPNB/C | unknown |
| Aizoon | Aizoaceae | 0.498041 | Wadi el-Jilat 13 | S Levant | PPNB/C | unknown |
| Aizoon | Aizoaceae | 0.457146 | Wadi el-Jilat 13 | S Levant | PPNB/C | unknown |
| Aizoon | Aizoaceae | 0.453271 | Wadi el-Jilat 13 | S Levant | PPNB/C | unknown |
| Aizoon | Aizoaceae | 0.375301 | Wadi el-Jilat 13 | S Levant | PPNB/C | unknown |
| Aizoon | Aizoaceae | 0.371094 | Wadi el-Jilat 13 | S Levant | PPNB/C | unknown |
| Aizoon hispanicum | Aizoaceae | 0.541349 | El Kowm I | N Levant | PPNB/C | unknown |
| Amaranthaceae | Amaranthaceae | 0.450996 | Çatalhöyük East | Central Turkey | PPNB/C | External firespot |
| Atriplex | Amaranthaceae | 0.72614 | Dj'ade | N Levant | PPNB/C | Midden |
| Atriplex | Amaranthaceae | 0.64416 | Dj'ade | N Levant | PPNB/C | Internal |
| Atriplex | Amaranthaceae | 0.519233 | Dj'ade | N Levant | PPNB/C | unknown |
| Atriplex | Amaranthaceae | 0.488758 | Dj'ade | N Levant | PPNB/C | unknown |
| Atriplex | Amaranthaceae | 0.428165 | Dj'ade | N Levant | PPNB/C | Internal |
| Chenopodium | Amaranthaceae | 0.450996 | Çatalhöyük East | Central Turkey | PPNB/C | External firespot |
| Chenopodium | Amaranthaceae | 0.433564 | Çatalhöyük East | Central Turkey | PPNB/C | External firespot |
| Suaeda | Amaranthaceae | 0.787879 | El Kowm II | N Levant | PPNB/C | External |
| Pistacia | Anacardiaceae | 0.620143 | Zahrat adh-Dhra 2 | S Levant | Pre/PPNA | unknown |
| Pistacia | Anacardiaceae | 0.611175 | Zahrat adh-Dhra 2 | S Levant | Pre/PPNA | unknown |
| Pistacia | Anacardiaceae | 0.606533 | Çayönü | N Levant | PPNB/C | Pit |
| Pistacia | Anacardiaceae | 0.604918 | Jerf el Ahmar | N Levant | Pre/PPNA | External firespot |
| Pistacia | Anacardiaceae | 0.578626 | Zahrat adh-Dhra 2 | S Levant | Pre/PPNA | unknown |
| Pistacia | Anacardiaceae | 0.537117 | Dj'ade | N Levant | PPNB/C | unknown |
| Pistacia | Anacardiaceae | 0.527472 | Zahrat adh-Dhra 2 | S Levant | Pre/PPNA | unknown |
| Pistacia | Anacardiaceae | 0.517968 | Çayönü | N Levant | PPNB/C | Pit |
| Pistacia | Anacardiaceae | 0.506255 | Zahrat adh-Dhra 2 | S Levant | Pre/PPNA | unknown |
| Lactuca | Asteraceae | 0.529438 | Hallan Çemi | E Fertile Crescent | Pre/PPNA | External |
| Lactuca | Asteraceae | 0.524885 | Hallan Çemi | E Fertile Crescent | Pre/PPNA | External |
| Arnebia decumbens | Boraginaceae | 0.601953 | El Kowm I | N Levant | PPNB/C | unknown |
| Arnebia decumbens | Boraginaceae | 0.600284 | El Kowm I | N Levant | PPNB/C | unknown |
| Arnebia decumbens | Boraginaceae | 0.599386 | El Kowm I | N Levant | PPNB/C | unknown |
| Arnebia decumbens | Boraginaceae | 0.597312 | Abu Hureyra | N Levant | PPNB/C | unknown |
| Arnebia decumbens | Boraginaceae | 0.568951 | Abu Hureyra | N Levant | PPNB/C | Internal |
| Arnebia decumbens | Boraginaceae | 0.568759 | Abu Hureyra | N Levant | PPNB/C | Internal |
| Arnebia decumbens | Boraginaceae | 0.566294 | Abu Hureyra | N Levant | PPNB/C | unknown |
| Arnebia decumbens | Boraginaceae | 0.564505 | Abu Hureyra | N Levant | PPNB/C | Pit |
| Arnebia decumbens | Boraginaceae | 0.546092 | El Kowm II | N Levant | PPNB/C | Pit |
| Arnebia decumbens | Boraginaceae | 0.533962 | Abu Hureyra | N Levant | PPNB/C | Pit |
| Arnebia decumbens | Boraginaceae | 0.513284 | Abu Hureyra | N Levant | PPNB/C | Internal |
| Arnebia decumbens | Boraginaceae | 0.474036 | Abu Hureyra | N Levant | PPNB/C | Internal |
| Arnebia decumbens | Boraginaceae | 0.473103 | Abu Hureyra | N Levant | PPNB/C | Internal |
| Arnebia decumbens | Boraginaceae | 0.42559 | Abu Hureyra | N Levant | PPNB/C | unknown |
| Arnebia decumbens | Boraginaceae | 0.422957 | Abu Hureyra | N Levant | PPNB/C | Internal |
| Buglossoides arvensis | Boraginaceae | 0.865082 | Asikli Höyük | Central Turkey | PPNB/C | Internal |
| Buglossoides arvensis | Boraginaceae | 0.776614 | Can Hasan III | Central Turkey | PPNB/C | unknown |
| Buglossoides arvensis | Boraginaceae | 0.636515 | Can Hasan III | Central Turkey | PPNB/C | unknown |
| Buglossoides arvensis | Boraginaceae | 0.604578 | Can Hasan III | Central Turkey | PPNB/C | unknown |
| Buglossoides tenuiflora | Boraginaceae | 0.639779 | Tell Bouqras | N Levant | PPNB/C | Internal |
| Alyssum/Lepidium | Brassicaceae | 0.428165 | Dj'ade | N Levant | PPNB/C | Internal |
| Brassica/Sinapis | Brassicaceae | 0.879251 | Jerf el Ahmar | N Levant | Pre/PPNA | Internal |
| Brassicaceae | Brassicaceae | 1 | Çatalhöyük East | Central Turkey | PPNB/C | Burnt destruction deposit |
| Brassicaceae | Brassicaceae | 1 | Çatalhöyük East | Central Turkey | PPNB/C | Burnt destruction deposit |
| Brassicaceae | Brassicaceae | 1 | Çatalhöyük East | Central Turkey | PPNB/C | Burnt destruction deposit |
| Brassicaceae | Brassicaceae | 1 | Çatalhöyük East | Central Turkey | PPNB/C | Burnt destruction deposit |
| Brassicaceae | Brassicaceae | 0.999929 | Çatalhöyük East | Central Turkey | PPNB/C | Burnt destruction deposit |
| Brassicaceae | Brassicaceae | 0.999899 | Çatalhöyük East | Central Turkey | PPNB/C | Burnt destruction deposit |
| Brassicaceae | Brassicaceae | 0.99951 | Çatalhöyük East | Central Turkey | PPNB/C | Burnt destruction deposit |
| Brassicaceae | Brassicaceae | 0.999429 | Çatalhöyük East | Central Turkey | PPNB/C | Burnt destruction deposit |
| Brassicaceae | Brassicaceae | 0.9988 | Çatalhöyük East | Central Turkey | PPNB/C | Burnt destruction deposit |
| Brassicaceae | Brassicaceae | 0.995831 | Çatalhöyük East | Central Turkey | PPNB/C | Burnt destruction deposit |
| Brassicaceae | Brassicaceae | 0.995805 | Çatalhöyük East | Central Turkey | PPNB/C | Burnt destruction deposit |
| Brassicaceae | Brassicaceae | 0.995177 | Çatalhöyük East | Central Turkey | PPNB/C | Burnt destruction deposit |
| Brassicaceae | Brassicaceae | 0.994987 | Çatalhöyük East | Central Turkey | PPNB/C | Burnt destruction deposit |
| Brassicaceae | Brassicaceae | 0.994247 | Çatalhöyük East | Central Turkey | PPNB/C | Burnt destruction deposit |
| Brassicaceae | Brassicaceae | 0.98048 | Çatalhöyük East | Central Turkey | PPNB/C | Burnt destruction deposit |
| Brassicaceae | Brassicaceae | 0.9524 | Çatalhöyük East | Central Turkey | PPNB/C | Burnt destruction deposit |
| Brassicaceae | Brassicaceae | 0.948554 | Çatalhöyük East | Central Turkey | PPNB/C | Burnt destruction deposit |
| Brassicaceae | Brassicaceae | 0.947767 | Çatalhöyük East | Central Turkey | PPNB/C | Burnt destruction deposit |
| Brassicaceae | Brassicaceae | 0.930002 | Çatalhöyük East | Central Turkey | PPNB/C | Internal |
| Brassicaceae | Brassicaceae | 0.927211 | Çatalhöyük East | Central Turkey | PPNB/C | Burnt destruction deposit |
| Brassicaceae | Brassicaceae | 0.925573 | Çatalhöyük East | Central Turkey | PPNB/C | Burnt destruction deposit |
| Brassicaceae | Brassicaceae | 0.892368 | Çatalhöyük East | Central Turkey | PPNB/C | Burnt destruction deposit |
| Brassicaceae | Brassicaceae | 0.860602 | Çatalhöyük East | Central Turkey | PPNB/C | Burnt destruction deposit |
| Brassicaceae | Brassicaceae | 0.839615 | Çatalhöyük East | Central Turkey | PPNB/C | Burnt destruction deposit |
| Brassicaceae | Brassicaceae | 0.796729 | Çatalhöyük East | Central Turkey | PPNB/C | Burnt destruction deposit |
| Brassicaceae | Brassicaceae | 0.775876 | Çatalhöyük East | Central Turkey | PPNB/C | Burnt destruction deposit |
| Brassicaceae | Brassicaceae | 0.720272 | Çatalhöyük East | Central Turkey | PPNB/C | Burnt destruction deposit |
| Brassicaceae | Brassicaceae | 0.681767 | Tell Abr | N Levant | Pre/PPNA | Internal |
| Brassicaceae | Brassicaceae | 0.656607 | Çatalhöyük East | Central Turkey | PPNB/C | Burnt destruction deposit |
| Brassicaceae | Brassicaceae | 0.610999 | Çatalhöyük East | Central Turkey | PPNB/C | Burnt destruction deposit |
| Brassicaceae | Brassicaceae | 0.574394 | Çatalhöyük East | Central Turkey | PPNB/C | Burnt destruction deposit |
| Brassicaceae | Brassicaceae | 0.571377 | Çatalhöyük East | Central Turkey | PPNB/C | Burnt destruction deposit |
| Brassicaceae | Brassicaceae | 0.547411 | Çatalhöyük East | Central Turkey | PPNB/C | Burnt destruction deposit |
| Brassicaceae | Brassicaceae | 0.511027 | Çatalhöyük East | Central Turkey | PPNB/C | Burnt destruction deposit |
| Brassicaceae | Brassicaceae | 0.488051 | Çatalhöyük East | Central Turkey | PPNB/C | Burnt destruction deposit |
| Brassicaceae | Brassicaceae | 0.472414 | Çatalhöyük East | Central Turkey | PPNB/C | Burnt destruction deposit |
| Brassicaceae | Brassicaceae | 0.449181 | Çatalhöyük East | Central Turkey | PPNB/C | Burnt destruction deposit |
| Brassicaceae | Brassicaceae | 0.423297 | Jerf el Ahmar | N Levant | Pre/PPNA | External |
| Brassicaceae | Brassicaceae | 0.387375 | Jerf el Ahmar | N Levant | Pre/PPNA | External |
| Capsella | Brassicaceae | 0.931495 | Çatalhöyük East | Central Turkey | PPNB/C | Vessel |
| Descurainia | Brassicaceae | 1 | Çatalhöyük East | Central Turkey | PPNB/C | Vessel |
| Descurainia | Brassicaceae | 1 | Çatalhöyük East | Central Turkey | PPNB/C | Vessel |
| Descurainia | Brassicaceae | 0.999712 | Çatalhöyük East | Central Turkey | PPNB/C | Vessel |
| Descurainia sophia | Brassicaceae | 0.999769 | Çatalhöyük East | Central Turkey | PPNB/C | Vessel |
| Descurainia sophia | Brassicaceae | 0.997965 | Çatalhöyük East | Central Turkey | PPNB/C | Burnt destruction deposit |
| Descurainia sophia | Brassicaceae | 0.99478 | Çatalhöyük East | Central Turkey | PPNB/C | Internal |
| Descurainia sophia | Brassicaceae | 0.988229 | Çatalhöyük East | Central Turkey | PPNB/C | Burnt destruction deposit |
| Descurainia sophia | Brassicaceae | 0.963156 | Çatalhöyük East | Central Turkey | PPNB/C | Internal hearth or oven |
| Descurainia sophia | Brassicaceae | 0.931929 | Çatalhöyük East | Central Turkey | PPNB/C | Vessel |
| Descurainia sophia | Brassicaceae | 0.928663 | Çatalhöyük East | Central Turkey | PPNB/C | Internal hearth or oven |
| Descurainia sophia | Brassicaceae | 0.92685 | Çatalhöyük East | Central Turkey | PPNB/C | Burnt destruction deposit |
| Descurainia sophia | Brassicaceae | 0.917979 | Çatalhöyük East | Central Turkey | PPNB/C | Internal hearth or oven |
| Descurainia sophia | Brassicaceae | 0.910386 | Çatalhöyük East | Central Turkey | PPNB/C | Burnt destruction deposit |
| Descurainia sophia | Brassicaceae | 0.897064 | Çatalhöyük East | Central Turkey | PPNB/C | Burnt destruction deposit |
| Descurainia sophia | Brassicaceae | 0.823754 | Çatalhöyük East | Central Turkey | PPNB/C | Internal hearth or oven |
| Descurainia sophia | Brassicaceae | 0.7945 | Çatalhöyük East | Central Turkey | PPNB/C | Burnt destruction deposit |
| Descurainia sophia | Brassicaceae | 0.667286 | Çatalhöyük East | Central Turkey | PPNB/C | Burnt destruction deposit |
| Descurainia sophia | Brassicaceae | 0.636408 | Çatalhöyük East | Central Turkey | PPNB/C | Internal hearth or oven |
| Descurainia sophia | Brassicaceae | 0.429537 | Çatalhöyük East | Central Turkey | PPNB/C | Vessel |
| Descurainia sophia | Brassicaceae | 0.401793 | Çatalhöyük East | Central Turkey | PPNB/C | Internal hearth or oven |
| Descurainia sophia | Brassicaceae | 0.378924 | Çatalhöyük East | Central Turkey | PPNB/C | Internal hearth or oven |
| Sisymbrium | Brassicaceae | 0.768922 | Çatalhöyük East | Central Turkey | PPNB/C | Vessel |
| Capparis | Capparaceae | 0.548752 | Tell Ras Shamra | N Levant | PPNB/C | unknown |
| Helianthemum | Cistaceae | 0.995789 | Çatalhöyük East | Central Turkey | PPNB/C | Vessel |
| Helianthemum | Cistaceae | 0.69065 | Gritille | N Levant | PPNB/C | External firespot |
| Helianthemum | Cistaceae | 0.416396 | Çatalhöyük East | Central Turkey | PPNB/C | Midden |
| Helianthemum ledifolium | Cistaceae | 0.596904 | Tell Ramad | S Levant | PPNB/C | unknown |
| Bolboschoenus glaucus | Cyperaceae | 0.468615 | Çatalhöyük East | Central Turkey | PPNB/C | Vessel |
| Bolboschoenus glaucus | Cyperaceae | 0.422162 | Çatalhöyük East | Central Turkey | PPNB/C | unknown |
| Bolboschoenus maritimus | Cyperaceae | 0.719467 | Demirkoy | E Fertile Crescent | Pre/PPNA | unknown |
| Bolboschoenus maritimus | Cyperaceae | 0.707244 | Hallan Çemi | E Fertile Crescent | Pre/PPNA | External firespot |
| Bolboschoenus maritimus | Cyperaceae | 0.583179 | Çatalhöyük East | Central Turkey | PPNB/C | Midden |
| Bolboschoenus maritimus | Cyperaceae | 0.579464 | Demirkoy | E Fertile Crescent | Pre/PPNA | Internal |
| Bolboschoenus maritimus | Cyperaceae | 0.549735 | Çatalhöyük East | Central Turkey | PPNB/C | Midden |
| Bolboschoenus maritimus | Cyperaceae | 0.521067 | Çatalhöyük East | Central Turkey | PPNB/C | External firespot |
| Bolboschoenus maritimus | Cyperaceae | 0.511513 | Hallan Çemi | E Fertile Crescent | Pre/PPNA | External |
| Bolboschoenus maritimus | Cyperaceae | 0.49791 | Hallan Çemi | E Fertile Crescent | Pre/PPNA | Internal |
| Bolboschoenus maritimus | Cyperaceae | 0.497545 | Tell Bouqras | N Levant | PPNB/C | Internal hearth or oven |
| Bolboschoenus maritimus | Cyperaceae | 0.495868 | Çatalhöyük East | Central Turkey | PPNB/C | Pit |
| Bolboschoenus maritimus | Cyperaceae | 0.486464 | Hallan Çemi | E Fertile Crescent | Pre/PPNA | External |
| Bolboschoenus maritimus | Cyperaceae | 0.480085 | Hallan Çemi | E Fertile Crescent | Pre/PPNA | External |
| Bolboschoenus maritimus | Cyperaceae | 0.476333 | Çatalhöyük East | Central Turkey | PPNB/C | unknown |
| Bolboschoenus maritimus | Cyperaceae | 0.433564 | Çatalhöyük East | Central Turkey | PPNB/C | External firespot |
| Bolboschoenus maritimus | Cyperaceae | 0.419308 | Çatalhöyük East | Central Turkey | PPNB/C | Midden |
| Bolboschoenus maritimus | Cyperaceae | 0.389823 | Çatalhöyük East | Central Turkey | PPNB/C | External firespot |
| Bolboschoenus maritimus | Cyperaceae | 0.385791 | Çatalhöyük East | Central Turkey | PPNB/C | Midden |
| Bolboschoenus maritimus | Cyperaceae | 0.3819 | Çatalhöyük East | Central Turkey | PPNB/C | Midden |
| Bolboschoenus maritimus/Eleocharis dulcis | Cyperaceae | 0.47686 | Abu Hureyra | N Levant | Pre/PPNA | Pit |
| Bolboschoenus maritimus/Eleocharis dulcis | Cyperaceae | 0.461861 | Abu Hureyra | N Levant | Pre/PPNA | Pit |
| Bolboschoenus maritimus/Eleocharis dulcis | Cyperaceae | 0.401832 | Abu Hureyra | N Levant | Pre/PPNA | Pit |
| Bolboschoenus maritimus/Eleocharis dulcis | Cyperaceae | 0.392533 | Abu Hureyra | N Levant | Pre/PPNA | Internal |
| Carex | Cyperaceae | 0.395207 | Çatalhöyük East | Central Turkey | PPNB/C | External firespot |
| Scirpus | Cyperaceae | 0.414164 | Sheikh-e Abad | E Fertile Crescent | PPNB/C | External |
| Astragalus | Fabaceae | 0.596904 | Tell Ramad | S Levant | PPNB/C | unknown |
| Astragalus | Fabaceae | 0.490442 | Tell Ramad | S Levant | PPNB/C | unknown |
| Astragalus/Trigonella | Fabaceae | 0.549735 | Çatalhöyük East | Central Turkey | PPNB/C | Midden |
| Astragalus/Trigonella | Fabaceae | 0.538287 | Çatalhöyük East | Central Turkey | PPNB/C | Midden |
| Astragalus/Trigonella | Fabaceae | 0.433504 | Çatalhöyük East | Central Turkey | PPNB/C | Midden |
| Fabaceae | Fabaceae | 0.498471 | Nevali Çori | N Levant | PPNB/C | unknown |
| Fabaceae | Fabaceae | 0.466524 | Nevali Çori | N Levant | PPNB/C | unknown |
| Fabaceae | Fabaceae | 0.437466 | Abu Hureyra | N Levant | PPNB/C | unknown |
| Fabaceae | Fabaceae | 0.419801 | Dj'ade | N Levant | PPNB/C | Internal |
| Fabeae | Fabaceae | 0.402027 | Mlefaat | E Fertile Crescent | Pre/PPNA | Internal |
| Lathyrus | Fabaceae | 0.783964 | Gritille | N Levant | PPNB/C | Midden |
| Lens | Fabaceae | 0.983399 | Çatalhöyük East | Central Turkey | PPNB/C | Vessel |
| Lens | Fabaceae | 0.659356 | Cafer Höyük | N Levant | PPNB/C | unknown |
| Lens | Fabaceae | 0.655147 | Jericho | S Levant | PPNB/C | Pit |
| Lens | Fabaceae | 0.557998 | Dj'ade | N Levant | PPNB/C | Midden |
| Lens | Fabaceae | 0.521438 | Dj'ade | N Levant | PPNB/C | unknown |
| Lens | Fabaceae | 0.52081 | Dj'ade | N Levant | PPNB/C | unknown |
| Lens | Fabaceae | 0.52038 | Çayönü | N Levant | PPNB/C | Internal |
| Lens | Fabaceae | 0.515911 | Dj'ade | N Levant | PPNB/C | unknown |
| Lens | Fabaceae | 0.507638 | Çatalhöyük East | Central Turkey | PPNB/C | unknown |
| Lens | Fabaceae | 0.393952 | Dj'ade | N Levant | PPNB/C | unknown |
| Lens culinaris | Fabaceae | 0.989321 | Çatalhöyük East | Central Turkey | PPNB/C | Vessel |
| Lens culinaris | Fabaceae | 0.698111 | Hacilar | Central Turkey | PPNB/C | Vessel |
| Lens culinaris | Fabaceae | 0.399006 | Çatalhöyük East | Central Turkey | PPNB/C | Vessel |
| Melilotus | Fabaceae | 0.583104 | Tell Ghoraifé | S Levant | PPNB/C | unknown |
| Pisum elatius | Fabaceae | 0.909936 | Hacilar | Central Turkey | PPNB/C | Vessel |
| Pisum elatius | Fabaceae | 0.69802 | Hacilar | Central Turkey | PPNB/C | Burnt destruction deposit |
| Pisum sativum | Fabaceae | 0.735871 | Çatalhöyük East | Central Turkey | PPNB/C | Burnt destruction deposit |
| Pisum sativum | Fabaceae | 0.723496 | Çatalhöyük East | Central Turkey | PPNB/C | Burnt destruction deposit |
| Pisum sativum | Fabaceae | 0.570171 | Nevali Çori | N Levant | PPNB/C | unknown |
| Pisum/Vicia/Lathyrus | Fabaceae | 0.687943 | Chogha Bonut | E Fertile Crescent | PPNB/C | unknown |
| Pisum/Vicia/Lathyrus | Fabaceae | 0.466098 | Dj'ade | N Levant | PPNB/C | Midden |
| Pisum/Vicia/Lathyrus | Fabaceae | 0.427648 | Dj'ade | N Levant | PPNB/C | unknown |
| SSLEG | Fabaceae | 0.647252 | Abu Hureyra | N Levant | PPNB/C | Internal |
| SSLEG | Fabaceae | 0.644478 | Wadi el-Jilat 13 | S Levant | PPNB/C | unknown |
| SSLEG | Fabaceae | 0.625552 | Abu Hureyra | N Levant | PPNB/C | Internal |
| SSLEG | Fabaceae | 0.615941 | Abu Hureyra | N Levant | PPNB/C | External firespot |
| SSLEG | Fabaceae | 0.601889 | Çatalhöyük East | Central Turkey | PPNB/C | External firespot |
| SSLEG | Fabaceae | 0.583305 | Abu Hureyra | N Levant | PPNB/C | Internal |
| SSLEG | Fabaceae | 0.568951 | Abu Hureyra | N Levant | PPNB/C | Internal |
| SSLEG | Fabaceae | 0.557767 | Abu Hureyra | N Levant | PPNB/C | Internal |
| SSLEG | Fabaceae | 0.5384 | Wadi el-Jilat 13 | S Levant | PPNB/C | unknown |
| SSLEG | Fabaceae | 0.533856 | El Kowm II | N Levant | PPNB/C | Internal hearth or oven |
| SSLEG | Fabaceae | 0.532913 | El Kowm II | N Levant | PPNB/C | Internal |
| SSLEG | Fabaceae | 0.527821 | Abu Hureyra | N Levant | PPNB/C | Internal |
| SSLEG | Fabaceae | 0.526981 | Abu Hureyra | N Levant | PPNB/C | unknown |
| SSLEG | Fabaceae | 0.526039 | El Kowm II | N Levant | PPNB/C | Internal hearth or oven |
| SSLEG | Fabaceae | 0.520622 | Wadi el-Jilat 7 | S Levant | PPNB/C | unknown |
| SSLEG | Fabaceae | 0.513284 | Abu Hureyra | N Levant | PPNB/C | Internal |
| SSLEG | Fabaceae | 0.512253 | El Kowm II | N Levant | PPNB/C | Internal hearth or oven |
| SSLEG | Fabaceae | 0.503612 | Abu Hureyra | N Levant | PPNB/C | Internal |
| SSLEG | Fabaceae | 0.503528 | Wadi el-Jilat 7 | S Levant | PPNB/C | Internal |
| SSLEG | Fabaceae | 0.494517 | Abu Hureyra | N Levant | PPNB/C | Internal |
| SSLEG | Fabaceae | 0.473103 | Abu Hureyra | N Levant | PPNB/C | Internal |
| SSLEG | Fabaceae | 0.467438 | Abu Hureyra | N Levant | PPNB/C | Internal |
| SSLEG | Fabaceae | 0.462436 | Wadi el-Jilat 7 | S Levant | PPNB/C | unknown |
| SSLEG | Fabaceae | 0.455318 | Çatalhöyük East | Central Turkey | PPNB/C | Internal hearth or oven |
| SSLEG | Fabaceae | 0.447289 | Çatalhöyük East | Central Turkey | PPNB/C | External firespot |
| SSLEG | Fabaceae | 0.422985 | Wadi el-Jilat 7 | S Levant | PPNB/C | unknown |
| SSLEG | Fabaceae | 0.415492 | Çatalhöyük East | Central Turkey | PPNB/C | Midden |
| SSLEG | Fabaceae | 0.397117 | Wadi el-Jilat 13 | S Levant | PPNB/C | unknown |
| Trigonella astroites | Fabaceae | 0.536326 | Tell Aswad | S Levant | PPNB/C | unknown |
| Trigonella astroites | Fabaceae | 0.457906 | Tell Aswad | S Levant | Pre/PPNA | unknown |
| Vicia | Fabaceae | 0.600348 | Çayönü | N Levant | PPNB/C | Internal |
| Vicia | Fabaceae | 0.596068 | Çayönü | N Levant | PPNB/C | Internal |
| Vicia ervilia | Fabaceae | 0.806356 | Çayönü | N Levant | PPNB/C | Internal |
| Vicia ervilia | Fabaceae | 0.787305 | Çayönü | N Levant | PPNB/C | Internal |
| Vicia ervilia | Fabaceae | 0.786589 | Çayönü | N Levant | PPNB/C | Internal |
| Vicia ervilia | Fabaceae | 0.783634 | Çayönü | N Levant | PPNB/C | Internal |
| Vicia ervilia | Fabaceae | 0.777651 | Çayönü | N Levant | PPNB/C | Internal |
| Vicia ervilia | Fabaceae | 0.777347 | Gritille | N Levant | PPNB/C | unknown |
| Vicia ervilia | Fabaceae | 0.748286 | Çayönü | N Levant | PPNB/C | Internal |
| Vicia ervilia | Fabaceae | 0.648931 | Çayönü | N Levant | PPNB/C | Internal |
| Vicia/Lathyrus | Fabaceae | 0.605642 | Çatalhöyük East | Central Turkey | PPNB/C | Internal |
| Juncus | Juncaceae | 0.599115 | Çatalhöyük East | Central Turkey | PPNB/C | External firespot |
| Juncus | Juncaceae | 0.545518 | Çatalhöyük East | Central Turkey | PPNB/C | External firespot |
| Juncus | Juncaceae | 0.54457 | Çatalhöyük East | Central Turkey | PPNB/C | Burnt destruction deposit |
| Juncus | Juncaceae | 0.469252 | Çatalhöyük East | Central Turkey | PPNB/C | External firespot |
| Juncus | Juncaceae | 0.405528 | Çatalhöyük East | Central Turkey | PPNB/C | External firespot |
| Ziziphora | Lamiaceae | 0.538872 | Tell Qaramel | N Levant | Pre/PPNA | unknown |
| Linum usitatissimum | Linaceae | 0.639758 | Tell Sabi Abyad II | N Levant | PPNB/C | unknown |
| Malva sylvestris | Malvaceae | 0.559093 | Cafer Höyük | N Levant | PPNB/C | External firespot |
| Ficus | Moraceae | 0.806377 | El Kowm I | N Levant | PPNB/C | unknown |
| Ficus | Moraceae | 0.596711 | El Kowm I | N Levant | PPNB/C | unknown |
| Aeluropus | Poaceae | 0.679468 | Çatalhöyük East | Central Turkey | PPNB/C | External firespot |
| Aeluropus | Poaceae | 0.490066 | Çatalhöyük East | Central Turkey | PPNB/C | External firespot |
| Aeluropus | Poaceae | 0.430721 | Çatalhöyük East | Central Turkey | PPNB/C | External firespot |
| Agrostis | Poaceae | 0.592802 | Ganj Dareh Tepe | E Fertile Crescent | PPNB/C | unknown |
| Agrostis | Poaceae | 0.583968 | Ganj Dareh Tepe | E Fertile Crescent | PPNB/C | unknown |
| Alopecurus | Poaceae | 0.675698 | Çatalhöyük East | Central Turkey | PPNB/C | unknown |
| Avena sterilis | Poaceae | 0.823223 | Gilgal | S Levant | Pre/PPNA | Internal |
| Bromus | Poaceae | 0.521995 | Çatalhöyük East | Central Turkey | PPNB/C | External firespot |
| Cereal indeterminate | Poaceae | 0.660692 | Çatalhöyük East | Central Turkey | PPNB/C | External firespot |
| Cereal indeterminate | Poaceae | 0.646369 | Ais Yiorkis | Cyprus | PPNB/C | Pit |
| Cereal indeterminate | Poaceae | 0.604655 | Tell Bouqras | N Levant | PPNB/C | Internal |
| Cereal indeterminate | Poaceae | 0.599753 | Tell Aswad | S Levant | PPNB/C | unknown |
| Cereal indeterminate | Poaceae | 0.595872 | Tell Aswad | S Levant | PPNB/C | unknown |
| Cereal indeterminate | Poaceae | 0.589647 | Tell Bouqras | N Levant | PPNB/C | Internal |
| Cereal indeterminate | Poaceae | 0.582662 | Nevali Çori | N Levant | PPNB/C | unknown |
| Cereal indeterminate | Poaceae | 0.578693 | Tell Ramad | S Levant | PPNB/C | unknown |
| Cereal indeterminate | Poaceae | 0.573409 | Ais Yiorkis | Cyprus | PPNB/C | Pit |
| Cereal indeterminate | Poaceae | 0.557949 | Tell Bouqras | N Levant | PPNB/C | Internal |
| Cereal indeterminate | Poaceae | 0.556089 | Tell Aswad | S Levant | PPNB/C | unknown |
| Cereal indeterminate | Poaceae | 0.551272 | Tell Aswad | S Levant | PPNB/C | unknown |
| Cereal indeterminate | Poaceae | 0.539559 | el-Hemmeh | S Levant | PPNB/C | Midden |
| Cereal indeterminate | Poaceae | 0.539536 | Ais Yiorkis | Cyprus | PPNB/C | Pit |
| Cereal indeterminate | Poaceae | 0.515793 | Tell Ramad | S Levant | PPNB/C | unknown |
| Cereal indeterminate | Poaceae | 0.507369 | Tell Aswad | S Levant | PPNB/C | unknown |
| Cereal indeterminate | Poaceae | 0.500632 | Ais Yiorkis | Cyprus | PPNB/C | Pit |
| Cereal indeterminate | Poaceae | 0.48404 | Tell Aswad | S Levant | PPNB/C | unknown |
| Cereal indeterminate | Poaceae | 0.476869 | Tell Aswad | S Levant | PPNB/C | unknown |
| Cereal indeterminate | Poaceae | 0.468789 | Tell Ramad | S Levant | PPNB/C | unknown |
| Cereal indeterminate | Poaceae | 0.464144 | Tell Aswad | S Levant | PPNB/C | unknown |
| Cereal indeterminate | Poaceae | 0.449835 | Tell Aswad | S Levant | PPNB/C | unknown |
| Cereal indeterminate | Poaceae | 0.448559 | Tell Aswad | S Levant | PPNB/C | unknown |
| Cereal indeterminate | Poaceae | 0.447375 | Tell Aswad | S Levant | PPNB/C | unknown |
| Cereal indeterminate | Poaceae | 0.43803 | Çatalhöyük East | Central Turkey | PPNB/C | Midden |
| Cereal indeterminate | Poaceae | 0.436276 | Tell Ramad | S Levant | PPNB/C | unknown |
| Cereal indeterminate | Poaceae | 0.433021 | Tell Ramad | S Levant | PPNB/C | unknown |
| Cereal indeterminate | Poaceae | 0.429537 | Çatalhöyük East | Central Turkey | PPNB/C | Vessel |
| Cereal indeterminate | Poaceae | 0.4258 | Tell Ramad | S Levant | PPNB/C | unknown |
| Cereal indeterminate | Poaceae | 0.424579 | Tell Ramad | S Levant | PPNB/C | unknown |
| Cereal indeterminate | Poaceae | 0.414154 | Tell Ramad | S Levant | PPNB/C | unknown |
| Cereal indeterminate | Poaceae | 0.403612 | Tell Ramad | S Levant | PPNB/C | unknown |
| Cereal indeterminate | Poaceae | 0.401881 | Çatalhöyük East | Central Turkey | PPNB/C | Midden |
| Cereal indeterminate | Poaceae | 0.401343 | Tell Ramad | S Levant | PPNB/C | unknown |
| Cereal indeterminate | Poaceae | 0.390862 | Çatalhöyük East | Central Turkey | PPNB/C | Midden |
| Cereal indeterminate | Poaceae | 0.389918 | Tell Ramad | S Levant | PPNB/C | unknown |
| Cereal indeterminate | Poaceae | 0.376703 | Tell Aswad | S Levant | PPNB/C | unknown |
| Crypsis | Poaceae | 0.597154 | Çatalhöyük East | Central Turkey | PPNB/C | External firespot |
| Crypsis aculeata | Poaceae | 0.398771 | Çatalhöyük East | Central Turkey | PPNB/C | Internal hearth or oven |
| Hordeum (non-cereal) | Poaceae | 0.434134 | Abu Hureyra | N Levant | PPNB/C | unknown |
| Hordeum (non-cereal) | Poaceae | 0.395747 | Abu Hureyra | N Levant | PPNB/C | Internal |
| Hordeum murinum/bulbosum | Poaceae | 0.655986 | Jerf el Ahmar | N Levant | Pre/PPNA | Internal |
| Hordeum murinum/bulbosum | Poaceae | 0.638337 | Jerf el Ahmar | N Levant | Pre/PPNA | Midden |
| Hordeum murinum/bulbosum | Poaceae | 0.614463 | Jerf el Ahmar | N Levant | Pre/PPNA | Midden |
| Hordeum spontaneum | Poaceae | 0.852757 | Jerf el Ahmar | N Levant | Pre/PPNA | Internal |
| Hordeum spontaneum | Poaceae | 0.688304 | Dj'ade | N Levant | PPNB/C | unknown |
| Hordeum spontaneum | Poaceae | 0.621838 | Jerf el Ahmar | N Levant | Pre/PPNA | unknown |
| Hordeum spontaneum | Poaceae | 0.575329 | Jerf el Ahmar | N Levant | Pre/PPNA | unknown |
| Hordeum spontaneum | Poaceae | 0.532859 | Jerf el Ahmar | N Levant | Pre/PPNA | Internal |
| Hordeum spontaneum | Poaceae | 0.522113 | Jerf el Ahmar | N Levant | Pre/PPNA | unknown |
| Hordeum spontaneum | Poaceae | 0.515414 | Jerf el Ahmar | N Levant | Pre/PPNA | unknown |
| Hordeum spontaneum | Poaceae | 0.512104 | Dj'ade | N Levant | PPNB/C | unknown |
| Hordeum spontaneum | Poaceae | 0.508563 | Jerf el Ahmar | N Levant | Pre/PPNA | unknown |
| Hordeum spontaneum | Poaceae | 0.505417 | Jerf el Ahmar | N Levant | Pre/PPNA | Internal |
| Hordeum spontaneum | Poaceae | 0.505254 | Jerf el Ahmar | N Levant | Pre/PPNA | unknown |
| Hordeum spontaneum | Poaceae | 0.503171 | Jerf el Ahmar | N Levant | Pre/PPNA | Midden |
| Hordeum spontaneum | Poaceae | 0.484603 | Jerf el Ahmar | N Levant | Pre/PPNA | Internal |
| Hordeum spontaneum | Poaceae | 0.47819 | Jerf el Ahmar | N Levant | Pre/PPNA | Internal |
| Hordeum vulgare | Poaceae | 0.975512 | Hacilar | Central Turkey | PPNB/C | Burnt destruction deposit |
| Hordeum vulgare | Poaceae | 0.756805 | Hacilar | Central Turkey | PPNB/C | Vessel |
| Hordeum vulgare | Poaceae | 0.680199 | Yarym Tepe | E Fertile Crescent | PPNB/C | unknown |
| Hordeum vulgare distichum | Poaceae | 0.74682 | Jericho | S Levant | PPNB/C | unknown |
| Hordeum vulgare distichum | Poaceae | 0.641362 | Jericho | S Levant | PPNB/C | unknown |
| Hordeum vulgare hexastichum | Poaceae | 0.607784 | Hacilar | Central Turkey | PPNB/C | Burnt destruction deposit |
| Hordeum vulgare var. nudum | Poaceae | 0.802502 | Çatalhöyük East | Central Turkey | PPNB/C | Burnt destruction deposit |
| Hordeum vulgare var. nudum | Poaceae | 0.794886 | Çatalhöyük East | Central Turkey | PPNB/C | Burnt destruction deposit |
| Hordeum vulgare var. nudum | Poaceae | 0.791458 | Çatalhöyük East | Central Turkey | PPNB/C | Burnt destruction deposit |
| Hordeum vulgare var. nudum | Poaceae | 0.766659 | Çatalhöyük East | Central Turkey | PPNB/C | Burnt destruction deposit |
| Hordeum vulgare var. nudum | Poaceae | 0.732521 | Çatalhöyük East | Central Turkey | PPNB/C | Burnt destruction deposit |
| Hordeum vulgare var. nudum | Poaceae | 0.655033 | Çatalhöyük East | Central Turkey | PPNB/C | Burnt destruction deposit |
| Hordeum vulgare var. nudum | Poaceae | 0.613374 | Yarym Tepe | E Fertile Crescent | PPNB/C | unknown |
| Hordeum vulgare var. nudum | Poaceae | 0.499599 | Çatalhöyük East | Central Turkey | PPNB/C | Burnt destruction deposit |
| Hordeum vulgare var. nudum | Poaceae | 0.423412 | Çatalhöyük East | Central Turkey | PPNB/C | Burnt destruction deposit |
| Hordeum vulgare var. nudum/spontaneum | Poaceae | 0.664797 | Yarym Tepe | E Fertile Crescent | PPNB/C | Vessel |
| Hordeum vulgare/spontaneum | Poaceae | 0.823223 | Çatalhöyük East | Central Turkey | PPNB/C | Burnt destruction deposit |
| Hordeum vulgare/spontaneum | Poaceae | 0.503703 | Çatalhöyük East | Central Turkey | PPNB/C | Burnt destruction deposit |
| Hordeum vulgare/spontaneum | Poaceae | 0.502737 | Ganj Dareh Tepe | E Fertile Crescent | PPNB/C | unknown |
| Lolium | Poaceae | 0.501087 | Mylouthkia | Cyprus | PPNB/C | Pit |
| Lolium | Poaceae | 0.442922 | Mylouthkia | Cyprus | PPNB/C | unknown |
| Lolium perenne/rigidum | Poaceae | 0.521918 | Kastros | Cyprus | PPNB/C | unknown |
| Poa | Poaceae | 0.855708 | Sheikh-e Abad | E Fertile Crescent | PPNB/C | unknown |
| Poa | Poaceae | 0.766861 | Sheikh-e Abad | E Fertile Crescent | PPNB/C | Internal |
| Poa | Poaceae | 0.721411 | Sheikh-e Abad | E Fertile Crescent | PPNB/C | Internal |
| Poa | Poaceae | 0.702338 | Sheikh-e Abad | E Fertile Crescent | PPNB/C | unknown |
| Poa | Poaceae | 0.689805 | Sheikh-e Abad | E Fertile Crescent | PPNB/C | Internal |
| Poa | Poaceae | 0.637561 | Sheikh-e Abad | E Fertile Crescent | PPNB/C | unknown |
| Poa | Poaceae | 0.626239 | Sheikh-e Abad | E Fertile Crescent | PPNB/C | External firespot |
| Poa | Poaceae | 0.586372 | Sheikh-e Abad | E Fertile Crescent | PPNB/C | Internal |
| Poaceae | Poaceae | 0.550126 | Dj'ade | N Levant | PPNB/C | unknown |
| Poaceae | Poaceae | 0.542012 | Tell Abr | N Levant | Pre/PPNA | unknown |
| Poaceae | Poaceae | 0.501087 | Mylouthkia | Cyprus | PPNB/C | Pit |
| Poaceae | Poaceae | 0.495352 | Gritille | N Levant | PPNB/C | External |
| Poaceae (small-seeded) | Poaceae | 0.657529 | Chogha Golan | E Fertile Crescent | PPNB/C | unknown |
| Poaceae (small-seeded) | Poaceae | 0.552161 | Gritille | N Levant | PPNB/C | Pit |
| Poaceae (small-seeded) | Poaceae | 0.48466 | Gritille | N Levant | PPNB/C | Midden |
| Sporobolus | Poaceae | 0.443646 | Çatalhöyük East | Central Turkey | PPNB/C | Midden |
| Taeniatherum | Poaceae | 0.422309 | Dj'ade | N Levant | PPNB/C | unknown |
| Taeniatherum | Poaceae | 0.390429 | Dj'ade | N Levant | PPNB/C | unknown |
| Taeniatherum caput-medusae | Poaceae | 0.858905 | Çatalhöyük East | Central Turkey | PPNB/C | Burnt destruction deposit |
| Taeniatherum caput-medusae | Poaceae | 0.845999 | Çatalhöyük East | Central Turkey | PPNB/C | Burnt destruction deposit |
| Triticum | Poaceae | 0.569533 | Çatalhöyük East | Central Turkey | PPNB/C | Burnt destruction deposit |
| Triticum | Poaceae | 0.517862 | Çatalhöyük East | Central Turkey | PPNB/C | Burnt destruction deposit |
| Triticum | Poaceae | 0.499599 | Çatalhöyük East | Central Turkey | PPNB/C | Burnt destruction deposit |
| Triticum aestivum/durum | Poaceae | 0.701505 | Tell Bouqras | N Levant | PPNB/C | Internal |
| Triticum aestivum/durum | Poaceae | 0.656607 | Çatalhöyük East | Central Turkey | PPNB/C | Burnt destruction deposit |
| Triticum aestivum/durum | Poaceae | 0.652096 | Tell Bouqras | N Levant | PPNB/C | Internal |
| Triticum aestivum/durum | Poaceae | 0.640895 | Çatalhöyük East | Central Turkey | PPNB/C | Burnt destruction deposit |
| Triticum aestivum/durum | Poaceae | 0.609905 | Tell Bouqras | N Levant | PPNB/C | Internal |
| Triticum aestivum/durum | Poaceae | 0.605508 | Tell Bouqras | N Levant | PPNB/C | Internal |
| Triticum aestivum/durum | Poaceae | 0.604655 | Tell Bouqras | N Levant | PPNB/C | Internal |
| Triticum aestivum/durum | Poaceae | 0.589647 | Tell Bouqras | N Levant | PPNB/C | Internal |
| Triticum aestivum/durum | Poaceae | 0.582295 | Tell Bouqras | N Levant | PPNB/C | Internal |
| Triticum aestivum/durum | Poaceae | 0.574394 | Çatalhöyük East | Central Turkey | PPNB/C | Burnt destruction deposit |
| Triticum aestivum/durum | Poaceae | 0.569533 | Çatalhöyük East | Central Turkey | PPNB/C | Burnt destruction deposit |
| Triticum aestivum/durum | Poaceae | 0.557949 | Tell Bouqras | N Levant | PPNB/C | Internal |
| Triticum boeoticum | Poaceae | 0.453936 | Tell Qaramel | N Levant | Pre/PPNA | unknown |
| Triticum boeoticum thaoudar | Poaceae | 0.839121 | Mureybet | N Levant | Pre/PPNA | unknown |
| Triticum boeoticum thaoudar | Poaceae | 0.762069 | Mureybet | N Levant | Pre/PPNA | unknown |
| Triticum boeoticum thaoudar | Poaceae | 0.608196 | Mureybet | N Levant | Pre/PPNA | unknown |
| Triticum boeoticum thaoudar | Poaceae | 0.583801 | Mureybet | N Levant | Pre/PPNA | unknown |
| Triticum boeoticum thaoudar | Poaceae | 0.581105 | Mureybet | N Levant | Pre/PPNA | unknown |
| Triticum boeoticum thaoudar | Poaceae | 0.580024 | Mureybet | N Levant | Pre/PPNA | unknown |
| Triticum boeoticum thaoudar | Poaceae | 0.549747 | Mureybet | N Levant | Pre/PPNA | unknown |
| Triticum dicoccum | Poaceae | 0.569284 | Yarym Tepe | E Fertile Crescent | PPNB/C | unknown |
| Triticum dicoccum/monococcum | Poaceae | 0.857518 | Çatalhöyük East | Central Turkey | PPNB/C | Burnt destruction deposit |
| Triticum dicoccum/monococcum | Poaceae | 0.827047 | Çatalhöyük East | Central Turkey | PPNB/C | Burnt destruction deposit |
| Triticum dicoccum/monococcum | Poaceae | 0.77232 | Çatalhöyük East | Central Turkey | PPNB/C | Burnt destruction deposit |
| Triticum dicoccum/monococcum | Poaceae | 0.75016 | Çatalhöyük East | Central Turkey | PPNB/C | Burnt destruction deposit |
| Triticum dicoccum/monococcum | Poaceae | 0.633623 | Çatalhöyük East | Central Turkey | PPNB/C | Burnt destruction deposit |
| Triticum dicoccum/monococcum | Poaceae | 0.609102 | Çatalhöyük East | Central Turkey | PPNB/C | Burnt destruction deposit |
| Triticum dicoccum/monococcum | Poaceae | 0.552768 | Çatalhöyük East | Central Turkey | PPNB/C | Burnt destruction deposit |
| Triticum dicoccum/monococcum | Poaceae | 0.504297 | Çatalhöyük East | Central Turkey | PPNB/C | Burnt destruction deposit |
| Triticum monococcum | Poaceae | 0.997691 | Hacilar | Central Turkey | PPNB/C | Burnt destruction deposit |
| Triticum monococcum | Poaceae | 0.590703 | Ais Yiorkis | Cyprus | PPNB/C | unknown |
| Triticum spelta | Poaceae | 0.538605 | Yarym Tepe | E Fertile Crescent | PPNB/C | unknown |
| Triticum/Secale | Poaceae | 0.827713 | Tell Abr | N Levant | Pre/PPNA | unknown |
| Triticum/Secale | Poaceae | 0.726855 | Tell Abr | N Levant | Pre/PPNA | unknown |
| Triticum/Secale | Poaceae | 0.653276 | Tell Abr | N Levant | Pre/PPNA | unknown |
| Triticum/Secale | Poaceae | 0.651443 | Tell Abr | N Levant | Pre/PPNA | unknown |
| Triticum/Secale | Poaceae | 0.608772 | Jerf el Ahmar | N Levant | Pre/PPNA | Internal hearth or oven |
| Triticum/Secale | Poaceae | 0.604346 | Tell Abr | N Levant | Pre/PPNA | unknown |
| Triticum/Secale | Poaceae | 0.541316 | Tell Qaramel | N Levant | Pre/PPNA | unknown |
| Triticum/Secale | Poaceae | 0.537762 | Tell Abr | N Levant | Pre/PPNA | unknown |
| Triticum/Secale | Poaceae | 0.506899 | Jerf el Ahmar | N Levant | Pre/PPNA | unknown |
| Polygonaceae | Polygonaceae | 0.577811 | Çatalhöyük East | Central Turkey | PPNB/C | Internal hearth or oven |
| Polygonum | Polygonaceae | 0.698195 | Mureybet | N Levant | Pre/PPNA | unknown |
| Polygonum | Polygonaceae | 0.580556 | Mureybet | N Levant | Pre/PPNA | unknown |
| Polygonum | Polygonaceae | 0.536491 | Mureybet | N Levant | Pre/PPNA | NA |
| Polygonum | Polygonaceae | 0.53208 | Mureybet | N Levant | Pre/PPNA | NA |
| Polygonum | Polygonaceae | 0.528325 | Mureybet | N Levant | Pre/PPNA | NA |
| Polygonum aviculare | Polygonaceae | 0.433756 | Çatalhöyük East | Central Turkey | PPNB/C | External firespot |
| Polygonum corrigioloides | Polygonaceae | 0.586528 | Abu Hureyra | N Levant | Pre/PPNA | Pit |
| Polygonum corrigioloides | Polygonaceae | 0.529272 | Abu Hureyra | N Levant | PPNB/C | Internal |
| Polygonum corrigioloides | Polygonaceae | 0.432794 | Abu Hureyra | N Levant | Pre/PPNA | Pit |
| Polygonum corrigioloides | Polygonaceae | 0.431411 | Abu Hureyra | N Levant | Pre/PPNA | Internal |
| Polygonum/Rumex | Polygonaceae | 0.534993 | Hallan Çemi | E Fertile Crescent | Pre/PPNA | Internal |
| Polygonum/Rumex | Polygonaceae | 0.510507 | Hallan Çemi | E Fertile Crescent | Pre/PPNA | External |
| Polygonum/Rumex | Polygonaceae | 0.470991 | Hallan Çemi | E Fertile Crescent | Pre/PPNA | Internal |
| Rumex | Polygonaceae | 0.577811 | Çatalhöyük East | Central Turkey | PPNB/C | Internal hearth or oven |
| Prunus | Rosaceae | 0.833694 | Tell Qaramel | N Levant | Pre/PPNA | unknown |
| Prunus | Rosaceae | 0.42303 | Jerf el Ahmar | N Levant | Pre/PPNA | Midden |
| Crucianella | Rubiaceae | 0.526039 | El Kowm II | N Levant | PPNB/C | Internal hearth or oven |
| Lycium | Solanaceae | 0.769514 | Çayönü | N Levant | PPNB/C | Internal |
| Lycium | Solanaceae | 0.62077 | Çayönü | N Levant | PPNB/C | Internal |
| Celtis | Ulmaceae | 0.777046 | Çatalhöyük East | Central Turkey | PPNB/C | unknown |
| Celtis | Ulmaceae | 0.766714 | Asikli Höyük | Central Turkey | PPNB/C | Internal |
| Celtis | Ulmaceae | 0.764689 | Asikli Höyük | Central Turkey | PPNB/C | Internal |
| Celtis | Ulmaceae | 0.734697 | Asikli Höyük | Central Turkey | PPNB/C | Internal |
| Celtis | Ulmaceae | 0.656536 | Asikli Höyük | Central Turkey | PPNB/C | Internal |
| Celtis | Ulmaceae | 0.573783 | Asikli Höyük | Central Turkey | PPNB/C | unknown |
| Celtis | Ulmaceae | 0.524555 | Asikli Höyük | Central Turkey | PPNB/C | Internal |
| Celtis | Ulmaceae | 0.522265 | Asikli Höyük | Central Turkey | PPNB/C | Internal |
| Celtis | Ulmaceae | 0.520762 | Asikli Höyük | Central Turkey | PPNB/C | unknown |
| Celtis | Ulmaceae | 0.415998 | Tell Qaramel | N Levant | Pre/PPNA | Pit |
| Celtis tournefortii | Ulmaceae | 0.604578 | Can Hasan III | Central Turkey | PPNB/C | unknown |

**Supplementary Equation A**

The identification of small and mixed samples was based on a calculated score (SM) similar to the DC score. The SM score was based on the distance from the origin (i.e. Simpson index of zero and normalised count of zero), normalised to a zero-to-one scale. Samples with an SM value <0.5 were taken to be small and mixed.

SM = SQRT ( (D)^2 + (C)^2) ) / SQRT (2)

**Supplementary Text A**

The database of sample-by-sample records that contributed to this paper from published sources is available is accessible online from the Archaeology Data Service. The database and further details about its contents and use can be found at doi.org/10.5284/1046750.
